# A Censored Mixture Model for Modeling Risk Taking

**DOI:** 10.1007/s11336-021-09839-1

**Published:** 2022-02-10

**Authors:** Nienke F. S. Dijkstra, Henning Tiemeier, Bernd Figner, Patrick J. F. Groenen

**Affiliations:** 1 Erasmus University Rotterdam, Rotterdam, The Netherlands; 2 HARVARD T.H. CHAN SCHOOL OF PUBLIC HEALTH, Boston, USA; 3 Radboud University, Behavioural Science Institute and Donders Institute for Brain, Cognition and Behaviour, Nijmegen, The Netherlands

**Keywords:** censoring, finite mixtures, multiple inflated model, Columbia Card Task, Generation R Study

## Abstract

Risk behavior has substantial consequences for health, well-being, and general behavior. The association between real-world risk behavior and risk behavior on experimental tasks is well documented, but their modeling is challenging for several reasons. First, many experimental risk tasks may end prematurely leading to censored observations. Second, certain outcome values can be more attractive than others. Third, a priori unknown groups of participants can react differently to certain risk-levels. Here, we propose the censored mixture model which models risk taking while dealing with censoring, attractiveness to certain outcomes, and unobserved individual risk preferences, next to experimental conditions.

**Supplementary Information:**

The online version contains supplementary material available at 10.1007/s11336-021-09839-1.

Taking a particular risk can have substantial consequences on health, well-being, and general behavior and, hence, is examined in many scientific fields, such as psychology, criminology, and economics. Risk taking can be measured by surveys or using experimental tasks. Although risk behavior across different experimental tasks does not highly correlate (Pedroni et al., 2017), studies have shown a moderate, but meaningful association between risk behavior measured in various experimental tasks and real-world risk taking. For example, Lejuez et al. (2003) and Prip , Neumann, Köhler, and Lamm (2013) show that smokers take significantly more risk on, respectively, the Balloon Analogue Risk Task (BART) and the Columbia Card Task (CCT) than non-smokers. Likewise, using a survey, Collins et al. (1987) show a relationship between risk taking/rebelliousness and smoking at an older age.

There are four types of experiments commonly used to measure risk behavior. The *first* is based on lotteries, where an explicit description of the outcome and probabilities are given. Typically, participants have to state their preference between, for example, option A: 50% chance of winning 10 euro, and option B: 30% chance of winning 30 euro. Typically, in these tasks it is straightforward to decompose the underlying constructs of risk taking. However, they are often criticized for being too artificial and lacking external validity.

An example of the *second* type of experiments is the Iowa Gambling Task (Bechara et al., 1994), where participants can win or lose money by picking (many) cards from four decks, each card having a win and loss value. The expected value and probability distribution of the values of the cards in the four decks are unknown to participants at the start, but can be learned during the task. This task has shown to successfully predict real-world risk taking behavior. However, it is virtually impossible to decompose the underlying constructs of participants’ risk taking behavior, such as risk preferences (Schonberg et al., 2011). Risk preferences are confounded with the learning curve, because participants have to unravel the expected return and the probability distribution of the decks while playing the game. In addition, it is difficult to distinguish whether the behavior is driven by risk attitude or sensitivity to reward or punishment.

The *third* type of task paradigm is based on gambling and includes, among others, the Cambridge Gambling Task (Rogers et al., 1999) and the Game of Dice Task (Brand et al., 2005), where participants have to bet on the color of randomly drawn cards or on the outcome of a roll of a dice, respectively. The probability of the possible outcomes is known, so there is no learning effect present. However, these tasks have the disadvantage that they do not allow to disentangle the effects of risk and of attractiveness of a higher expected pay-off value.

*Lastly*, in sequential risk tasks participants are asked to repeat a certain action. These tasks include, among others, the Balloon Analogue Risk Task (BART) (Lejuez et al., 2002), the Columbia Card Task (CCT) (Figner et al., 2009), the Angling Risk Task (Pleskac, 2008), and the Devil Task (Slovic, 1966), where the repeated actions are inflating a balloon, turning over cards, catching fish, and pulling knife switches, respectively. The risk increases the longer a participant continues. Although these sequential risk tasks do not suffer from the issues described under the first three types of experiments, they have their own challenges, which makes modeling risk taking complex. Below we discuss two of these challenges.

*First*, the analyses are often based on the assumption of a smooth (normal) distribution of the residuals. However, certain outcomes are more attractive to participants than others. For example, in some sequential risk tasks participants have to select a number of repetitions of a certain action, this number indicates the level of risk someone is prepared to take (e.g., the number of pumps in the BART, the number of cards turned over in the CCT, or the number of fish caught in the Angling Risk Task). It is well known that even numbers and multiples of five are more often selected than odd numbers (Baird et al., 1970). This pattern leads to inflated values in the outcome distribution. Similarly, within surveys some outcomes tend to be more attractive than others. Imagine a longitudinal study measuring drug usage. Asking people how often they use drugs, typically, also leads to even numbers and multiples of five and ten (Klesges et al., 1995).

The *second* challenge of sequential risk tasks concerns censored observations. In the imaginary longitudinal drug study, participants can be easily lost, leading to incomplete information and censored observations. Moreover, most sequential risk tasks by definition may randomly end prematurely, such as when a loss card is encountered in the CCT. Typically, the researcher is interested in the level of risk a participant is willing to take and the censoring obscures this.

To deal with censored observations, Lejuez et al. (2002) suggest to use the adjusted score in the BART (average inflations over the unpopped balloons). However, Pleskac et al. (2008) have shown that this score is biased and propose the automatic BART, where participants have to choose a number of inflations before the trial starts and censoring is no issue. Figner et al. (2009) argue that people behave differently in a preplanned (i.e., the automatic BART) and impulsive (i.e., the original BART) decision making situation and developed the Columbia Card Task (CCT) to investigate the difference between deliberative and affective decision making. The CCT is a computer-based card game, and participants can win or lose money by turning over cards. A major advantage of the CCT, over the other sequential risk tasks, is that the CCT orthogonally varies the risk-relevant factors gain amount, loss amount, and loss probability. Moreover, these factors are known to the participant, so the risk taking is not confounded with the effect of learning these values.

So far, none of the existing studies have provided a statistical model that addresses all the issues introduced above, that is censored observations, attractiveness to certain outcomes, and unobserved heterogeneity. However, there are two studies worth mentioning. *First*, Wallsten et al. (2005) introduce a learning model for the BART that accommodates censoring. This model, however, is not directly applicable to tasks where the probability of losing is known, like the CCT and some tournaments in the Angling Risk Task, because it is built on the fact that participants have prior believes about these probabilities and update their believes throughout the game. *Second*, Weller et al. (2019) implement an interesting model that addresses the censoring in the CCT and allows individual unobserved heterogeneity through a random effects model. A potential disadvantage of this model is the assumption on the distribution of the random effect. We propose a model that makes less parametric assumptions and models the unobserved heterogeneity in a more free way. We refer also to this paper for its excellent overview of the literature on risk decision making processes.

In the present study, we propose a censored mixture model (CMM). This model is a specific form of survival analyses that are used often in scientific studies in medicine, sociology, and econometrics to predict the duration of time until a specific event, such as death, cancer diagnosis, divorce, graduation from school, or finding a job. A common trait across these studies is that when the data collection ends, the event has occurred for some individuals, but not for others. For example, some couples divorce during the study, while others do not. These couples may divorce later, but, by the time the data are analyzed, it is unknown when. These incomplete observations are called censored observations. In our CMM, censored observations are included in the model by using the information that the participant intended to take more risk than the observed risk level. The attractiveness of certain patterns in outcome values is covered by assigning additional probability mass to the inflated values in the distribution. Furthermore, we choose to model the unobserved individual tendency for risk taking through finite mixtures, which can approximate a variety of distributions. In addition, we choose a link function such that the regression coefficients have a linear interpretation on the interval $[0, \inf \rangle$. In short, the CMM models risk taking while dealing with censoring, attractiveness to certain outcomes, and unobserved individual risk preferences, next to analyzing the experimental conditions and having a straightforward linear interpretation of the effects.

Our model can be used to analyze all sequential risk tasks. In the present study, we analyze the hot version of the CCT, which measures affective decision making. In addition, results of the CMM applied to a BART data set are added as Online Resource 1. The data of the CCT were collected by the Generation R Study which was designed to analyze early environmental and genetic determinants of growth, development, and health from fetal life until young adulthood. A total of 4538 nine-year-old children participated in the CCT.

The remainder of this paper is structured as follows. It starts with a detailed explanation of the CCT and its challenges when modeling risk taking. Next, the data are discussed by means of the data collection process, cleaning procedure, and their characteristics. Subsequently, the structure of the model is discussed extensively. Last, we present the results and we will discuss the limitations.

## Columbia Card Task

**Fig. 1:**
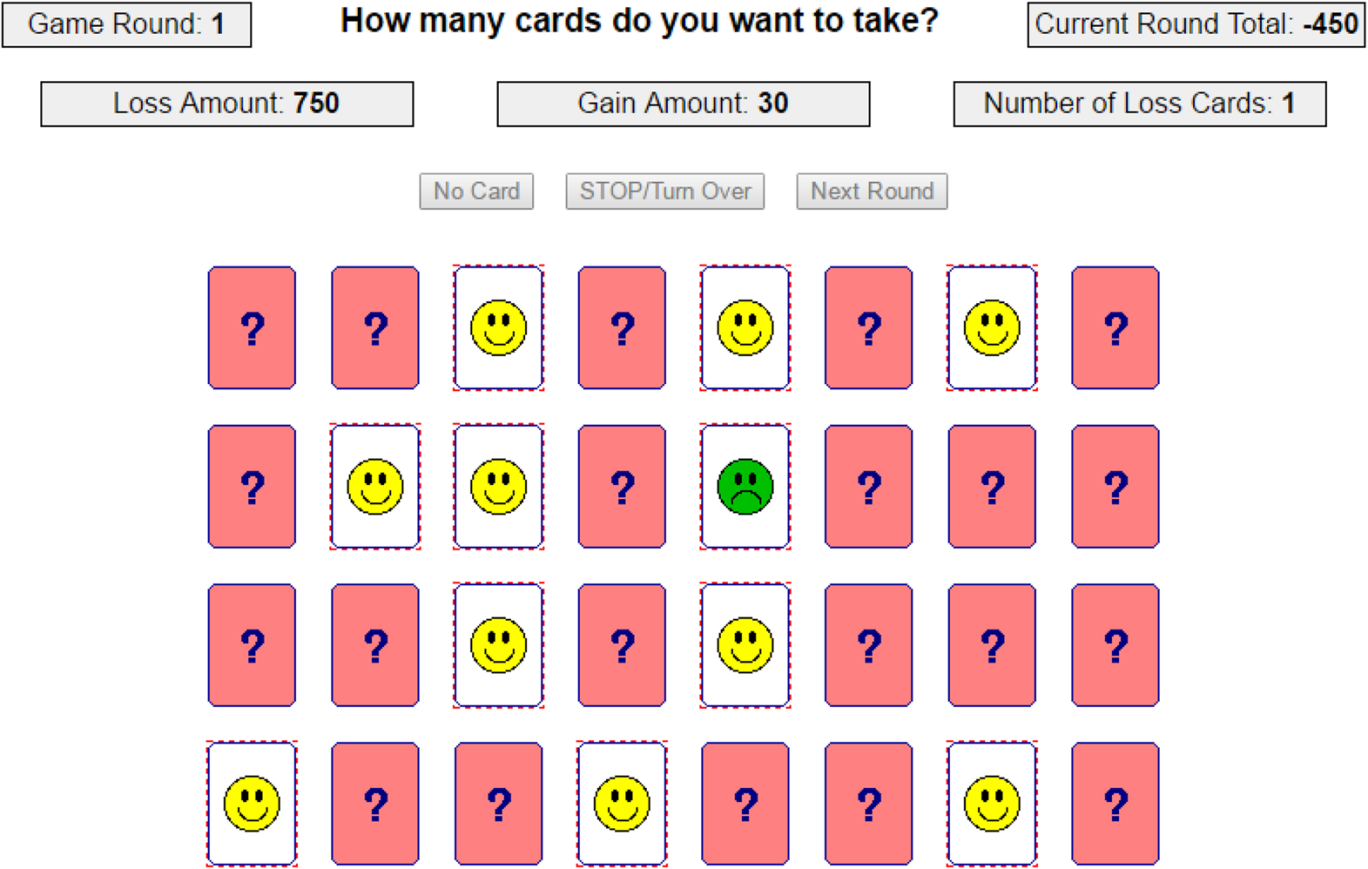
A screenshot of the first game round in the Columbia Card Task with the game settings: gain amount equal to thirty, loss amount equal to 750, and number of loss cards equal to one. In this game round, the participant first turned over ten win cards (happy faces). The eleventh card was a loss card (sad face), resulting in a total score in the current game round of $10 \times 30 - 750 = -450$.

The Columbia Card Task is shown in Fig. 1. There are 32 cards divided in win cards (happy faces) and loss cards (unhappy faces). At the beginning of a game round all cards are face down and participants are asked to turn over cards. By turning over a win card, the participant earns points and by turning over a loss card they lose points and the current game round ends. At every step, the participant has the choice between turning over another card and pressing the stop button to voluntarily stop this game round. It is also possible to stop immediately without turning over any card. After a game has ended, the earned points are summed and the potential loss amount is subtracted.

The gain amount (points earned by turning over a win card), loss amount (points lost by turning over a loss card), and number of loss cards vary per round: the gain amount is either ten or thirty, the loss amount is either 250 or 750, and there are either 1 or 3 loss cards in the game (Figner & Weber, 2011). These three experimental conditions are displayed at the top of the screen and are known to the participant. Note that, in contrast to Figner et al. (2009), the loss cards are randomly distributed over the 32 cards. These three parameters lead to eight different game settings and within a block of eight trials the sequence of the game settings is random. Every participant plays at least two blocks of eight trials^1^. In other words, every game setting is played at least twice. Because of the different game settings, the CCT measures next to risk taking also the complexity of information use and the sensitivity to reward, punishment, and probability. With the three parameters (gain amount, loss amount, and number of loss cards) is it possible to assess which of these three parameters affects participants’ choices.

The indicator for risk taking is the number of cards a participant intends to turn over. However, if a participant faces a loss card the game ends prematurely, the trial is censored and it is unknown how many cards a participant intended to turn over. This should be considered in the analysis. Figner et al. (2009) manipulate the game such that in most trials the loss card is the last possible card to turn over and only analyze the uncensored trials. However, for this manipulation not to be discovered by participants, Figner et al. (2009) included extra trials where the loss card appeared at an early stage of the trial. This approach has several drawbacks. Besides the serious problem that such a setup uses deception, letting participants play extra rounds has the important disadvantage of being time consuming and hence more expensive. In addition, we show that the result in the previous trial effects the behavior in the current trial. Not correcting for the negative shock a loss card might have, could affect the results.

**Fig. 2:**
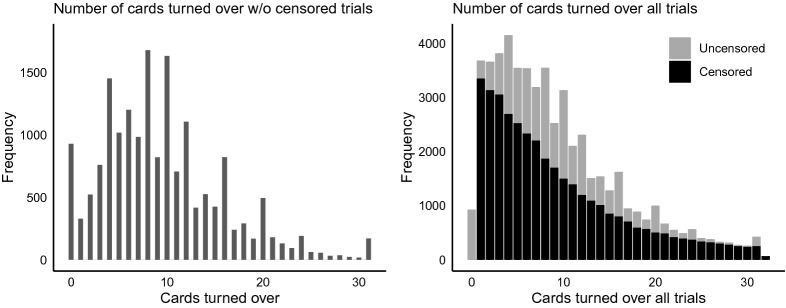
Distribution of the number of cards turned over.

Another issue that should be accounted for is the attractiveness of certain outcome values. Figure 2 shows the distribution of the outcome, in this case, the number of cards turned over. The left graph only includes the uncensored trials and shows peaks at certain number of cards. The right panel suggests that the peaks are independent of the probability of being censored, because the censored trials (lower bars) do not show any irregular or unexpected values. Three categories of peaks can be distinguished. The *first* category is the excess of zeros. This inflation is probably caused by children who are very much risk averse and prefer not to play the game. The *second* category includes the peaks at four, eight, ten, twelve, sixteen, twenty, and twenty-four. Although ten is not a multiple of four, it seems to be an attractive number similar to the multiples of four; hence, it is included in this set. Recall the layout of the CCT from Fig. 1, creating a geometric pattern, such as complete rows or columns, corresponds to turning over a number of cards equal to a multiple of four. The *third* category of excesses occurs with participants who are very risk seeking: if you managed to turn over 30 cards without facing a loss card, then why not as well try the 31^st^ card? Note that Categories 1, 2, and 3 are also subsumed in Category 4 and that the weight of the observation is equally split over Category 1, 2, or 3 and Category 4.

**Fig. 3:**
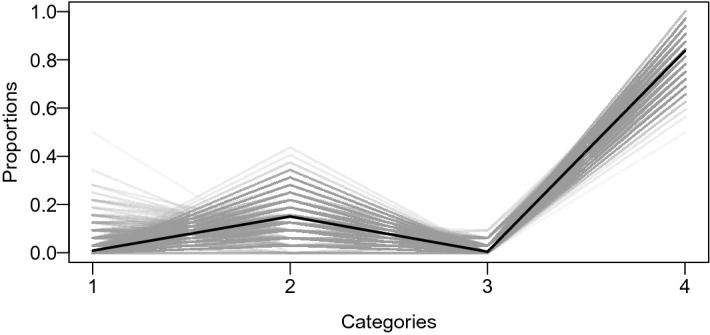
A parallel coordinates plot on proportions of outcomes in four categories: (1) zero cards turned over, (2) multiples of four, (3) 31 cards turned over, and (4) all possible outcomes (i.e., {0, 32}). Note that Categories 1, 2, and 3 are also subsumed in Category 4 and that the weight of the observation is equally split over Category 1, 2, or 3 and Category 4. The individual distributions are in gray, and the average over all distributions is in black.

To demonstrate that these categories of inflated values are independent over the individuals, we compute the distribution of the outcomes per person over four categories. Figure 3 provides a parallel coordinates plot with these individual distributions in gray and the average over all distributions in black. Strict independence would be found if the gray lines coincide with the mean line in black. Since the individual distributions are relatively similar, we argue that the inflated values are independent over the individuals.

## Data

What sets this research apart from previous studies, besides addressing all issues involved with modeling risk taking, is the large number of participants. The large sample size allows us to build a more flexible model that handles censoring, categorical background variables, such as individual characteristics, experimental conditions, and attractiveness to certain patterns and outcome values.

The current study is embedded in the Generation R Study, a large population based multi-ethnic cohort study (Kooijman et al., 2016). The Generation R Study was designed to analyze early environmental and genetic determinants of growth, development, and health from fetal life until young adulthood. The data collection is intense and includes multiple surveys with biological and observational assessments. The CCT is one of the observational assessments that was conducted on nine-year-old children (age 9.8 $\pm 0.26$). The cohort includes almost ten thousand children at birth, of which 4538 children participated in the CCT. The data set is partitioned in a training set of 3404 children and an (prior to analysis) unseen test set of 1134 children. The Generation R Study has an open policy in regard to collaboration with other research groups (http://www.generationr.nl/researchers/collaboration.html). Requests for data access and collaboration are discussed in the Generation R Study Management Team.

The CCT was conducted as part of a series of assessments taking approximately three hours. At the beginning of the CCT, children were told that they would be rewarded with money based on their performance on the CCT. After all trials were played, three trials were randomly selected and were paid out in real money. The children had a start value of 200 cents (i.e., 2 euro), and the total points of the selected trials were added or deducted from this start value. Children could receive money, but did not have to pay any net losses.

The prevalence of censoring (i.e., the number of observation with incomplete data) in this data set is $68\%$. Therefore, treating the censored observations as uncensored would lead to severe biases in the results. Available background variables include children’s age and IQ (102 $\pm 14.7$), measured with the SON-R 2.5-7 at the age of six. Furthermore, information about the mother is available in ethnicity (Dutch = 59.8%, Dutch Antilles = 2.1%, African and Moroccan $<5\%$, Asian (non-Western) and Turkish $<6\%$, Surinamese = 7.1%, and other Western = 10,1%) and education (low = 6.7%, middle = 42.2%, high = 51.2%). The last background variable is the household income per month in euros ($<2000 = 20.5\%$, $2000-4000 = 43.8\%$, $>4000 = 35.7\%$). Missing values in the background variables are imputed with single Predictive Mean Matching (PMM) using age, gender, weight at birth, and IQ of the child, and the age at delivery, ethnicity, and education of the mother, and household income as predictors, and using the mice package in R.

The segments obtained with the CMM will be interpreted using the child behavior checklist (CBCL). This survey assesses child emotional and behavioral problems as perceived by the mother. The CBCL has been completed at the same time as the CCT, at age nine. For some children, the CBCL scores at age nine were missing but available at age six. For these children, their scores at age nine are imputed by single PMM using the score at age six and the covariates in the model. The 223 children that have scores neither at age six nor at age nine are excluded from this analysis.

As discussed, the CMM can also be used to analyze other risk tasks, for example the BART. The Online Resource 1 contains the results of the CMM applied to a data set from a BART study (Dekkers et al., 2020).

## Methods

The following section is concerned with the methods and techniques applied in this study. First, the structure of the model is discussed, extensively. Although the model is applicable to all sequential risk tasks, we construct the likelihood function through the CCT.

### The Censored Mixture Model (CMM)

The CMM has its roots in the field of survival analysis. In the case of the CCT, we need to take care of three challenges: the censoring of the data, the attraction of particular outcomes, such as presented in Fig. 2, and unobserved heterogeneity across individuals. By constructing a likelihood function, we can explain step-by-step how we incorporate these challenges into the CMM. Additionally, we show explicitly that the censoring is exogenous (also known as independent or non-informative censoring), which is a key assumption in survival analysis. The possible censoring is accommodated by adding the cumulative distribution function, equivalent to the survival function, to the likelihood function. Extra probability mass is assigned to the inflated values in the outcome distribution. Last, the finite mixtures account for the unobserved individual characteristics. Apart from this, we follow a generalized linear model approach, that is, we will assume that there is a linear combination of covariates that provides, after transformation by a link function, the mean of a distribution for every observed number of cards. We argue that the negative binomial distribution is appropriate and provide a link function that is close to linear for ease of interpretation. Below, a step wise explanation is given how these potential problems are solved by the CMM.

The observed variable to be modeled is the number of cards turned over $y_{it}$ by individual *i* in trial *t*. Furthermore, we observe whether a trial is censored at card *k*, $c_{itk} = 1$, or not, $c_{itk} = 0$. However, we are interested in the latent random variable $Z_{it}$, indicating the number of cards someone intends to turn over. This variable is assumed to follow a known distribution (here we propose to use the negative binomial distribution). Now, the probability of the observed number of cards turned over can be expressed in terms of the latent random variable $Z_{it}$

$$\begin{aligned} \Pr (Y_{it} = k \wedge C_{itk} = c_{itk}) = \Pr (Y_{it} = k \wedge C_{itk} = c_{itk} \mid Z_{it} = \ell ) \Pr (Z_{it} = \ell ), \end{aligned}$$

where $C_{itk}$ indicates censoring at trial *t* for individual *i* at card *k* and $\ell$ is the intended number cards turned over.

More insight on the conditional probability $\Pr (Y_{it} = k \wedge C_{itk} = c_{itk} \mid Z_{it} = \ell )$ can be obtained by considering all possible outcomes of the game, that is, for all combinations of number of observed cards $y_{it}$, being censored or not ($c_{itk} = 1$ or 0), and the number of cards intended to turn over $z_{it}$. Table 1 provides these probabilities where the notation $p_k = \Pr (C_{itk} = 1 \mid C_{it\ell } = 0 \ \forall \ \ell < k)$ is used. Note that these probabilities are purely based on the game settings. Due to symmetry properties, this table can be summarized by

$$\begin{aligned} \Pr (Y_{it} = k \wedge C_{itk} = c_{itk} \mid Z_{it} = \ell )= & {} \left\{ \begin{aligned}&\Omega _{k\ell , c_{itk}} = \Omega _{kk, c_{itk}}&\forall \ell \ge k \text { if } c_{itk} = 1,\\&\Omega _{k\ell , c_{itk}} = 0 \ \ \ \&\forall \ell < k\text { if } c_{itk} = 1,\\&\Omega _{k\ell , c_{itk}} = 0 \ \ \ \&\forall \ell \ne k \text { if } c_{itk} = 0\\ \end{aligned} \right. \end{aligned}$$

where $\Omega _{k\ell , c_{itk}}$ corresponds to the fixed probabilities given in Table 1. Subsequently, the likelihood contribution for person *i* at trial *t* can be written as

$$\begin{aligned} \begin{aligned} L_{it}&= \sum _{\ell = 0}^{32} \Pr (Y_{it} = k \wedge C_{itk} = c_{itk} \mid Z_{it} = \ell ) \Pr (Z_{it}=\ell )\\&= \left\{ \begin{aligned}&\Omega _{k\ell , c_{itk}} \Pr (Z_{it} = \ell ) = \Omega _{k\ell , c_{itk}} \Theta _{\ell , c_{itk}}&\text {if } c_{itk} = 0 \\&\sum _{m = k}^{32} \Omega _{km, c_{itk}} \Pr (Z_{it} = m) = \Omega _{k\ell , c_{itk}} \sum _{m = k}^{32} \Pr (Z_{it} = m) = \Omega _{k\ell , c_{itk}} \Theta _{\ell , c_{itk}}&\text {if } c_{itk} = 1. \end{aligned}\right. \end{aligned} \end{aligned}$$

Thus, for $c_{itk} = 1$, $\Theta _{\ell , c_{itk}}$ equals one minus the cumulative distribution function, that is, $\Theta _{\ell , c_{itk}} = 1 - \sum _{\ell =0 }^{k-1} \Pr (Z_{it} = \ell )$ and for $c_{itk} = 0$ we have $\Theta _{\ell , c_{itk}} = \Pr (Z_{it} = \ell )$. Thus for $c_{itk} = 1$, $\Theta _{\ell , c_{itk}}$ is equivalent to the survival function. Multiplying over the trials, the likelihood contribution of person *i* can be written as

$$\begin{aligned} \begin{aligned} L_i&= \prod _{t = 1}^{T} \Omega _{y_{it}z_{it}, c_{itz_{it}}} \Theta _{z_{it}, c_{ity_{it}}}. \end{aligned} \end{aligned}$$

**Table 1:** The probability for each possible combination of the observed number of cards *k*, the intended number of cards $\ell$, and being censored at card *k* ($c_{itk}$), that is, $\Pr (Y_{it}~=~k \wedge C_{itk}~=~c_{itk} \mid Z_{it}~=~ \ell ) = \Omega _{k\ell , c_{itk}}$.

		$$z_{it} =\ell $$					
$y_{it} = k$	$c_{itk}$	0	1	2	$\dots$	31	32
0	0	1	0	0	$\dots$	0	0
0	1	0	0	0	$\dots$	0	0
1	0	0	$1-p_1$	0	$\dots$	0	0
1	1	0	$p_1$	$p_1$	$\dots$	$p_1$	$p_1$
2	0	0	0	$(1-p_1)(1-p_2)$	$\dots$	0	0
2	1	0	0	$(1-p_1)p_2$	$\dots$	$(1-p_1)p_2$	$(1-p_1)p_2$
$\vdots$	$\vdots$	$\vdots$	$\vdots$	$\vdots$	$\ddots$	$\vdots$	$\vdots$
31	0	0	0	0	$\dots$	$\prod _{i=1}^{31} (1-p_i)$	0
31	1	0	0	0	$\dots$	$\prod _{i=1}^{30} (1-p_i)p_{31}$	$\prod _{i=1}^{30} (1-p_i)p_{31}$
32	0	0	0	0	$\dots$	0	0
32	1	0	0	0	$\dots$	0	$\prod _{i=1}^{31} (1-p_i)p_{32}$

Although the probability $\Pr (Z_{it} = \ell )$ seems to follow a known distribution, it does not follow a smooth distribution, see Fig. 2. The left panel shows the number of cards a child intends to turn over, that is, proportionally the empirical equivalence of $\Pr (Y_{it} = k \mid C_{itk} = 0) = \Pr (Z_{it} = k)$. It is easy to see that some outcomes seem extra attractive. We choose to distinguish four of these cases: (a) $k = 0$, (b) $k \in A$ with $A = \{4, 8, 10, 12, 16, 20, 24\}$, (c) $k = 31$, and (d) otherwise. To control for these four cases, we implement a multiple inflation model, where the observations belonging to each of these cases get extra probability mass through parameter $\phi _m$ with $m = 1, \dots , 4$. The probability $\Pr (Z_{it} = \ell )$ is defined by

$$\begin{aligned} \Pr (Z_{it} = \ell ) = \left\{ \begin{array}{ll} \phi _4 f(0) + \phi _1 &{} \text {if } \ell = 0,\\ \phi _4 f(\ell ) + \frac{1}{|A|} \phi _2 &{} \text {if } \ell \in A,\\ \phi _4f(31) + \phi _3 &{} \text {if } \ell = 31,\\ \phi _4 (1-F(32)) &{} \text {if } \ell = 32,\\ \phi _4 f(\ell ) &{} \text {for all other values } \ell , \end{array} \right. \end{aligned}$$

where $f(\ell )$ and $F(\ell )$ are the probability mass function and cumulative distribution function, respectively, of a known distribution. Note that the weights $\phi _m$ have to be between zero and one, $0 \le \phi _m \le 1$, and sum to one, $\sum _{m=1}^{4} \phi _m = 1$.

Since the number of cards someone intends to turn over ($z_{it}$) is nonnegative and discrete, the distribution has to have these properties as well. We choose the negative binomial distribution, because it allows the variance to differ from the mean, in contrast to the Poisson distribution. The negative binomial distribution can be written as a Poisson–Gamma mixture. Specify the mean of the Poisson distribution as a combination of a deterministic function of the predictors, $\mu _{it} = g(\eta _{it})$, and a random component, $\nu \sim _{\text {i.i.d.}}g(\nu \mid \kappa )$. Let $g(\nu \mid \kappa )$ be the density of the Gamma distribution, then the resulting Poisson–Gamma mixture density can be rewritten as the negative binomial density. The probability mass function of this distribution is specified as follows:

$$\begin{aligned} f(z_{it} \mid \mu _{it}, \delta ) = \frac{\Gamma (\delta + z_{it})}{\Gamma (\delta ) z_{it}!} \left( \frac{\delta }{\delta + \mu _{it}}\right) ^{\delta } \left( \frac{\mu _{it}}{\mu _{it} + \delta }\right) ^{z_{it}}, \end{aligned}$$

where $$\delta = 1/\kappa $$.

In a generalized linear model (GLM), the mean $\mu _{it}$ of the distribution is specified through an inverse link function

$$\begin{aligned} \mu _{it} = h^{-1}(\eta _{it}). \end{aligned}$$

The mean $\mu _{it}$ of the negative binomial distribution must be larger than zero. Therefore, the inverse link function $h^{-1}(\eta _{it})$ should map $\eta _{it} \in {\mathbb {R}}$ to ${\mathbb {R}}^+$. In GLM, $\eta _{it}$ is chosen as a linear combination of covariates $\varvec{x}'_{it}$, that is,

$$\begin{aligned} \eta _{it} = \alpha + \varvec{x}'_{it} \varvec{\beta }. \end{aligned}$$

**Fig. 4:**
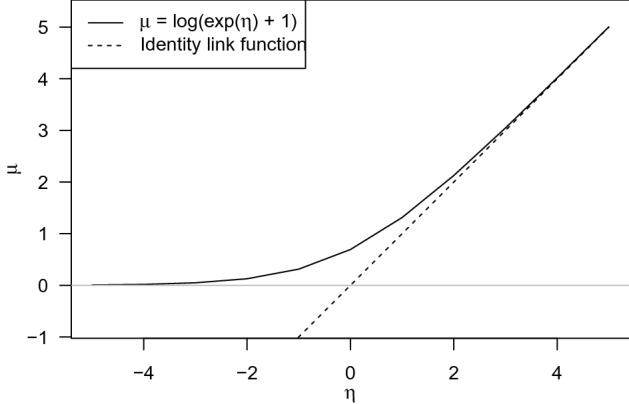
Both the proposed inverse link function, $\mu _{it} = h^{-1}(\eta _{it}) = \log (\exp (\eta _{it}) +1)$, and the identity link function, $\mu _{it} = h^{-1}(\eta _{it}) = \eta _{it}$, on the domain [$-5$:5].

For ease of interpretation of the coefficients $\varvec{\beta }$, we specify, the inverse link function by

$$\begin{aligned} h^{-1}(\eta _{it}) = \log (\exp (\eta _{it}) +1) \end{aligned}$$

so that whenever $\eta _{it} > 1$, the inverse link function becomes close to linear, yet $h^{-1}(\eta _{it}) > 0$ for any $\eta _{it}$, see Fig. 4 (see, e.g., Ranganath et al., 2016).

The predictor variables are all gathered in the vector $\varvec{x}_{it}$. Some predictor variables are categorical and we choose to represent each of them by their own dummy variable. Without loss of generality, the weights corresponding to the dummy variables for each categorical variable must have sum zero, that is,

$$\begin{aligned} \varvec{C\beta } = \varvec{0} \text{ with } \varvec{C} = \left[ \begin{array}{ccc} (\varvec{1'1})^{-1/2}\varvec{1}' &{} \varvec{0}' &{} \varvec{0}' \\ \varvec{0}' &{} (\varvec{1'1})^{-1/2}\varvec{1}' &{} \varvec{0}' \\ \end{array}\right] , \end{aligned}$$

where, for illustration, it is assumed that there are two categorical variables followed by numerical predictors, so that $\varvec{C}$ consists of one row per categorical variable with ones at the positions of the weights and zero elsewhere. Note that the factors $(\varvec{1'1})^{-1/2}$ are chosen for notational convenience. Again, without loss of generality, it is also assumed that the numerical predictor variables are *z*-scores (with mean zero and standard deviation one) so that the intercept $\alpha$ can be interpreted as an overall measure of risk taking for someone who has a neutral score on all predictors.

The complete likelihood over all *N* individuals becomes

$$\begin{aligned} L(\varvec{\theta })= & {} \prod _{i = 1}^{N} L_i = \prod _{i = 1}^{N} \prod _{t = 1}^{T} \Omega _{y_{it}z_{it}, c_{ity_{it}}} \Theta _{z_{it}, c_{ity_{it}}} \\= & {} \left( {\prod _{i = 1}^{N} \prod _{t = 1}^{T} \Omega _{y_{it}z_{it}, c_{ity_{it}}} }\right) \left( {\prod _{i = 1}^{N} \prod _{t = 1}^{T} \Theta _{z_{it}, c_{ity_{it}}} }\right) \\\propto & {} \prod _{i = 1}^{N} \prod _{t = 1}^{T} \Theta _{z_{it}, c_{ity_{it}}}, \end{aligned}$$

where $\varvec{\theta }$ is the vector of all unknown parameters. The factor $\prod _{i = 1}^{N} \prod _{t = 1}^{T} \Omega _{y_{it}z_{it}, c_{ity_{it}}}$ is irrelevant for maximizing the likelihood as it is constant, so that optimizing $\prod _{i = 1}^{N} \prod _{t = 1}^{T} \Theta _{z_{it}, c_{ity_{it}}}$ over $\varvec{\theta }$ is sufficient. Note that this independence implies that in the CMM the censoring is exogenous, meaning that the distribution of censoring does not provide information on the distribution of the number of cards turned over. We also assume that any carry-over effects are subsumed in the linear combination $\eta _{it}$, so that the conditional independence above still holds.

In a final step of the CMM, we wish to be able to model unobserved heterogeneity across individuals by adding finite mixtures with different intercepts per segment to the model, that is,

$$\begin{aligned} \eta _{its} = \alpha _s + \varvec{x}'_{it}\varvec{\beta }, \end{aligned}$$

with $\alpha _s$ the segment specific intercept. The relative size of the segment is estimated by $\pi _s$. Then, the likelihood function becomes

1 $$\begin{aligned} L(\varvec{\theta })= & {} \prod _{i = 1}^{N} \sum _{s = 1}^{S} \pi _s \prod _{t = 1}^{T} \Omega _{y_{it}z_{it}, c_{ity_{it}}} \prod _{t = 1}^{T} \Theta _{z_{it}, c_{ity_{it}},s}\nonumber \\= & {} \left( {\prod _{i = 1}^{N} \prod _{t = 1}^{T} \Omega _{y_{it}z_{it}, c_{ity_{it}}} }\right) \left( {\prod _{i = 1}^{N} \sum _{s = 1}^{S} \pi _s \prod _{t = 1}^{T} \Theta _{z_{it}, c_{ity_{it}},s} }\right) \nonumber \\\propto & {} \prod _{i = 1}^{N} \sum _{s = 1}^{S} \pi _s \prod _{t = 1}^{T} \Theta _{z_{it}, c_{ity_{it}},s}, \end{aligned}$$

where $\varvec{\theta }$ is understood to contain all unknown parameters. Note that $\Theta _{z_{it}, c_{ity_{it}},s}$ has obtained an additional subscript *s* to indicate that this probability is dependent on the parameter $\alpha _s$. Thus, the CMM needs to maximize $L(\varvec{\theta })$ over $\varvec{\theta } '= [\varvec{\alpha }', \varvec{\beta }', \delta , \varvec{\phi }', \varvec{\pi }']$ subject to $\varvec{C\beta } = \varvec{0}$, $\phi _m \ge 0$, $\varvec{1'\phi } = 1$, $\pi _s \ge 0$, and $\varvec{1'\pi } = 1$. More details about the estimation procedure can be found in Appendix  A.

## Results

Before the censored mixture model (CMM) can be applied to the data discussed in Sect. 3, several parameters need to be set. First, the maximization of the log likelihood function is performed through the optimx function in optimx package in R. All default settings are used except for the relative convergence tolerance, *reltol*, which is set more strictly such that the maximization has converged as soon as $\log L(\varvec{\theta }^{(t)})- \log L(\varvec{\theta }^{(t-1)})$ is less than $10^{-10} (\mid \log L(\varvec{\theta }^{(t)}) \mid + 10^{-10})$ where *t* is the iteration counter. After convergence, one step of the Newton-Raphson method is performed using a numerically approximated Hessian with the aim of ensuring that the gradient is close to zero. To speed up the convergence, the start values of $\varvec{\alpha }$ and $\varvec{\phi }$ are based on educated guesses. For $\varvec{\alpha }$, the start values are uniformly distributed over the possible outcomes, $\{0, 32\}$. The initial values of $\varvec{\phi }$ are based on the observed proportion of excesses in Fig. 2, that is, the difference of the observed proportion of the inflated outcome minus the interpolated value of the previous and next outcomes without inflation. To further improve the convergence speed, we trained our model on a small subsample, $n = 100$, and implemented these parameter estimates as start values of $\varvec{\theta }$ in the model using the original sample.

### Selection of the Number of Segments

**Fig. 5:**
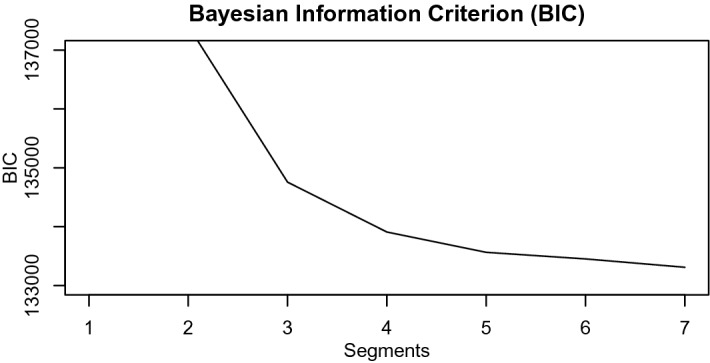
The Bayesian information criterion (BIC) of CMMs with $S = 2$ to 7 segments.

As the number of segments is unknown a priori, the model is computed for several numbers of segments *S*. We use several criteria to decide on a useful number of segments: the Bayesian information criterion (BIC), a minimum segment size, and the distinctiveness of the segment specific intercept. The BIC for various choices of *S* is shown in Fig. 5. Since the number of observations is so large in this study, adding a segment hardly affects the BIC. Therefore, searching for the number of segments that would lead to a minimum BIC would require an unrealistically high number of segments. Therefore, we additionally check the size of the segments $\pi _s$ and the segment specific intercepts $\alpha _s$ given in Table 2. We opt for segments that have $\pi _s > 5\%$ of the observations, that is, 170 children. Furthermore, we impose the segment specific intercepts $\alpha _s$ to be sufficiently different to avoid segments where the level of risk seeking as symbolized by their respective $\alpha _s$ is hardly different. Based on these three criteria, we choose to continue interpreting the model with $S = 4$ segments.

From Table 2, it is clear that Segment 1 is overall the smallest ($\pi _1 = 0.097$) and is characterized by children that are on average most risk averse as $\alpha _1$ is the smallest of all segments. In contrast, the last segment contains children that are most risk seeking as their intercept $\alpha _4 = 37.52$ is even larger than the total number of cards that could be turned over in the game.

**Table 2:** Segment probabilities $\pi _s$ and segment specific intercepts $\alpha _s$ with the standard errors between brackets for CMMs with $S = 2$ to 7 segments.

		Segment *s*						
*S*		1	2	3	4	5	6	7
2								
	$\pi _s$	0.394	0.606					
		(0.010)	(0.010)					
	$\alpha _s$	9.90	26.35					
		(0.164)	(0.313)					
3								
	$\pi _s$	0.150	0.432	0.419				
		(0.007)	(0.010)	(0.011)				
	$\alpha _s$	6.77	13.93	30.93				
		(0.148)	(0.184)	(0.408)				
4								
	$\pi _s$	0.097	0.275	0.357	0.271			
		(0.006)	(0.011)	(0.012)	(0.011)			
	$\alpha _s$	5.85	11.04	18.68	37.52			
		(0.152)	(0.188)	(0.295)	(0.772)			
5								
	$\pi _s$	0.023	0.119	0.284	0.331	0.243		
		(0.003)	(0.007)	(0.011)	(0.012)	(0.012)		
	$\alpha _s$	3.11	6.89	11.74	19.44	38.40		
		(0.167)	(0.148)	(0.187)	(0.322)	(0.904)		
6								
	$\pi _s$	0.002	0.103	0.206	0.256	0.249	0.164	
		(0.003)	(0.008)	(0.021)	(0.015)	(0.017)	(0.015)	
	$\alpha _s$	2.67	6.55	10.64	15.58	23.97	46.82	
		(0.258)	(0.168)	(0.315)	(0.587)	(0.852)	(2.685)	
7								
	$\pi _s$	0.007	0.052	0.130	0.294	0.303	0.000	0.214
		(0.002)	(0.005)	(0.009)	(0.012)	(0.012)	(0.000)	(0.013)
	$\alpha _s$	-0.66	5.03	8.36	12.99	21.07	22.70	40.83
		(0.225)	(0.163)	(0.188)	(0.225)	(0.438)	(148.3)	(1.328)

Note that the seven-segment solution is near the boundary and that $\alpha _6$ in this solution is poorly estimated.

### Segments Specific Results

**Fig. 6:**
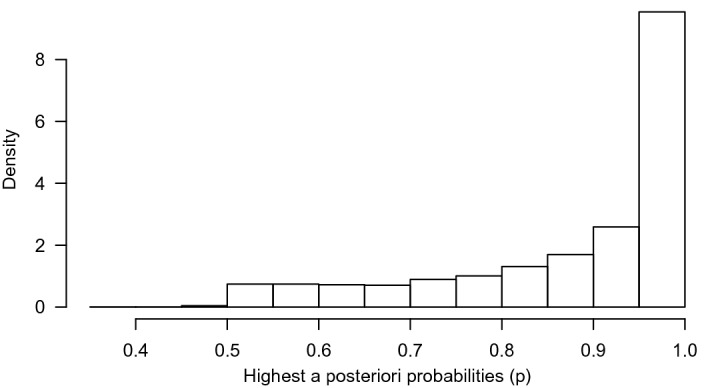
A histogram of the highest a posteriori segment probability of each individual.

For each individual, we can compute the a posteriori probability of belonging to a segment. Ideally, these probabilities are close to one for one of the segments and close to zero for the others thereby clearly assigning an individual to a segment. To see how distinctive the segments are, we consider the highest a posteriori probability for each individual and plot that in a histogram. Figure 6 shows this distribution and it is clear that indeed most children are assigned to a segment with a large probability. Therefore, virtually each child can be assigned with high probability to one of the segments.

It is interesting to investigate how the segments differ on characteristics that have not been part of the model. In particular, how do the segments differ with regard to the occurrence of behavioral problems as measured by the CBCL? The resulting *z*-scores (with mean zero and standard deviation one) per segment weighted by the a posteriori probabilities per segment are presented in Table 3. Appendix B discusses how to test for differences of weighted means. The stars in Table 3 denote whether one of the segment averages is significantly different from the overall average for this particular symptom.

The level of behavioral problems in all subscales except that of somatic complaints differs between the groups of children as defined by our segments. The risk averse children in Segment 1 and the risk seekers in Segment 4 on average have more behavioral problems than the children in Segment 2 and 3. Table 3 also suggests that children in Segment 3 who intend to turn over on average 18 cards score on average the lowest on all CBCL subscales. Furthermore, we can see from this table that a risk averse strategy is most profitable, as the average score is highest in Segment 1 (most risk averse segment) and lowest in Segment 4 (most risk seeking segment).

**Table 3:** Weighted *z*-scores per segment of CBCL subscales scores and other CCT characteristics.

	Segment *s*				
	1	2	3	4	Total
$\pi _s$	0.10	0.28	0.36	0.27	
CBCL subscales					
Internalizing ${^*}$	0.06	0.00	$-$0.05	0.05	0.00
Externalizing $${^{**}}$$	0.08	$$-$$0.03	$$-$$0.04	0.06	0.00
CBCL symptom subscales					
Anxiety ${^*}$	0.07	0.01	$-$0.06	0.04	0.00
Social withdrawal ${^*}$	0.02	0.02	$-$0.06	0.05	0.00
Somatic complaints	0.03	$-$0.02	$-$0.01	0.02	0.00
Social problems $${^{***}}$$	0.09	$$-$$0.04	$$-$$0.05	0.08	0.00
Thought problems $${^{**}}$$	0.10	$$-$$0.02	$$-$$0.05	0.05	0.00
Attention problems $${^{***}}$$	$$-$$0.03	$$-$$0.04	$$-$$0.06	0.13	0.00
Delinquent behavior $${^{**}}$$	0.00	$$-$$0.03	$$-$$0.03	0.08	0.00
Aggressive behavior ${^*}$	0.10	$-$0.02	$-$0.04	0.04	0.00
Average score $${^{***}}$$	$$-$$87.0	$$-$$123.7	$$-$$170.7	$$-$$230.1	$$-$$165.7
$$\#$$ cards turned over $${^{***}}$$	5.0	7.7	10.0	11.7	9.3
$$\#$$ censored trials $${^{***}}$$	5.8	8.5	11.4	14.3	10.8

A Wald test is performed to check for a significant difference between the segments. One star denotes $0.05 \le p <0.10$, two $0.01 \le p <0.05$, and three $p<0.01$. The 223 children without a CBCL score measured at either six or nine years old were excluded.

One of the contributions of the CMM model is that the intended number of cards to be turned over is estimated by the segment specific intercept $\alpha _s$. Due to the censoring, children who intend to turn over a high number of cards will often not be able to do so. Therefore, the observed number of cards turned over under estimates the intended number of cards to be turned over. We can easily compare them using the forelast row in Table 3 with the $\alpha _s$ from Table 2. For example, the average number of observed cards turned over by children in Segment 1 is 5.0 whereas average number of card intended is 5.9. For Segments 2, 3, and 4, these values are 7.7, 10.0, and 11.7 observed and 11.0, 18.7, and 37.5 intended. Indeed, a large under estimation of risk seeking is obtained when only considering the observed number of cards turned over.

### Regression Coefficients

The regression coefficients $\varvec{\beta }$ are presented in Table 4. The two numerical variables (age and IQ) are standardized to *z*-scores prior to the analysis. Whether or not a loss card was drawn in the last two games is recorded by the following variables: previous loss yes, previous loss no, second previous loss yes, and second previous loss no. As our link function in Fig. 4 is close to the identity function for values larger than 1, the coefficients can be interpreted on the scale of the number of cards turned over. For categorical variables, we chose mean weights of the categories belonging to a single variable to be zero so that the intercept can be interpreted as the average score in the segment for a neutral child. As a consequence, the difference in weights between two categories is the corresponding effect, for example, girls on average turn over $0.286 + \mid -0.286\mid = 0.572$ cards more than boys.

Furthermore, age and IQ have a negative association with the number of cards turned over. Also, a higher household income is related to higher levels of risk taking. Children with a mother with a Dutch or Asian ethnicity turn over fewer cards than the base average.

Due to the different game settings, we are able to investigate the effect of the loss probability and the sensitivity to reward and punishment. According to the model, the number of loss cards has the strongest effect on the number of cards turned over. In a game with three loss cards on average 1.7 cards less are turned over, than in a game with one loss card. The game setting loss amount also shows the expected direction of effect. In a trial with a high loss amount, the expected number of cards turned over is lower than in a trial with a low loss amount. Unexpectedly, the predicted number of cards turned over is lower in a trial with a high gain amount than it is in a trial with a low gain amount.

Moreover, the results in the previous round have a strong association with observed behavior in the current round. If a loss card was encountered in the previous round, on average 1.6 cards less are turned over. The experience of a loss card two trials earlier also relates to the intention to turn over one card less in the current trial. Note that these variables capture the immediate impact of a negative experience (e.g., turning over a loss card), not the learning effect. We argue that there is no learning effect in the current data set, since the average number of cards turned over per trial varies between 10.5 cards in trial 1 and 8.9 cards in trial 15.

We included interaction terms between the game settings and sex. According to the estimates, the combination of boy and a loss amount of 250 accounts for an additional 0.063 $(= -0.286 + 0.195 + 0.154)$ cards to be turned over. In a trial with loss amount 750, a boy is expected to turn over 0.635 $(= \mid -0.286 -0.195 -0.154\mid )$ cards less than the base average (i.e., the segment specific intercepts). Hence, the effect the loss amount has on the number of cards turned over by boys is 0.698 ($=0.063 + \mid -0.635 \mid$). This effect is smaller for girls, namely 0.572 ($=(0.286 +0.195 -0.154) + (0.286 - 0.195 + 0.154)$). Therefore, boys seem to be more sensitive to punishment in the CCT than girls are. Moreover, boys are also more influenced by the number of loss cards (2.038 vs. 1.360), whereas girls seem to be more sensitive to reward than boys are (0.346 vs. 1.026).

**Table 4:** Regression coefficients with their standard errors.

Background variables	$\beta$ - coefficients (st error)		Game settings	$\beta$ -coefficients (st error)	
Age	$-$0.012	(0.071)	Gain amount (10)	0.343	(0.039)
Boy	$-$0.286	(0.079)	Gain amount (30)	$-$0.343	(0.039)
Girl	0.286	(0.079)	Loss amount (250)	0.195	(0.038)
IQ	$-$0.539	(0.095)	Loss amount (750)	$-$0.195	(0.038)
Ethnicity mother			Loss cards (1)	0.850	(0.039)
Dutch	$-$1.170	(0.157)	Loss cards (3)	$-$0.850	(0.039)
Asian	$-$0.875	(0.258)	Previous loss yes	$-$0.823	(0.040)
African	0.570	(0.393)	Previous loss no	0.823	(0.040)
Moroccan	0.477	(0.338)	Second previous loss yes	$-$0.502	(0.040)
Dutch Antilles	$-$0.139	(0.379)	Second previous loss no	0.502	(0.040)
Surinamese	0.288	(0.378)	Interaction terms		
Turkish	0.527	(0.342)	Gain amount (10) : Boy	$-$0.170	(0.039)
Other Western	0.322	(0.265)	Gain amount (30) : Boy	0.170	(0.039)
Education mother			Gain amount (10) : Girl	0.170	(0.039)
No or primary	0.571	(0.219)	Gain amount (30) : Girl	$-$0.170	(0.039)
education					
Secondary education	$-$0.231	(0.143)	Loss amount (250) : Boy	0.154	(0.038)
Higher education	$-$0.340	(0.137)	Loss amount (750) : Boy	$-$0.154	(0.038)
Household income per month in euro’s			Loss amount (250) : Girl	$-$0.154	(0.038)
$< 2000$	$-$0.134	(0.163)	Loss amount (750) : Girl	0.154	(0.038)
$2000 - 4000$	$-$0.231	(0.114)	Loss cards (1) : Boy	0.169	(0.039)
$> 4000$	0.365	(0.123)	Loss cards (3) : Boy	$-$0.169	(0.039)
			Loss cards (1) : Girl	$-$0.169	(0.039)
			Loss cards (3) : Girl	0.169	(0.039)

Within a categorical variable the sum of coefficients sum to zero and the continues variables age and IQ are standardized.

### Model Performance

Our model gives of each child on each trial a probability distribution for the number of cards turned over. To obtain a sense how well the model fits the observed uncensored number of cards turned over, we compute a point estimate as the expected value of that distribution. Then, the model performance can be judged in terms of the difference between observed and expected number of cards turned over. Appendix C provides more details on how these expectations are computed. Predictions can be generated with our CMM. The in-sample root mean square error (RMSE) is equal to 8.5, and the mean absolute deviation (MAD) of the residuals is equal to 5.4. On a scale of 0–32 cards that can be turned over, these average deviations seem reasonable. To test the external validity of the model, the data set is randomly partitioned in a training set ($N = 3404$) and a prior to analysis unseen test set ($N = 1134$). The out-of-sample RMSE is equal to 8.4 and the MAD is equal to 5.3, showing little difference between in-sample and out-of-sample accuracy.

Another way to evaluate the model performance is by comparing the distributions of the empirical and predicted number of cards turned over for the training and the test data, similar to Fig. 2. We break down the comparison into a censored and uncensored case. For a fair comparison between the observed and predicted number of cards turned over, one has to multiply the distribution of the predicted outcome with the probability of being (un)censored. These probabilities can be derived from Table 1, where $p_k$ is equal to

$$\begin{aligned} \begin{aligned}&p_k = \frac{1}{32 - k - 1} \ \ \ \ \text { if }\#\text {loss cards } = 1\\&p_k = \frac{3}{32 - k - 1} \ \ \ \ \text { if }\#\text {loss cards } = 3. \end{aligned} \end{aligned}$$

The empirical probability of the number of cards intended to turn over in the training set is shown in the left panel of Fig. 8. The comparison with the right panel with the CMM predicted probabilities shows that these predictions are quite accurate. The left panel of Fig. 9 shows the empirical probability per card of being censored in the training data and the right panel shows these values as predicted. Again, the distribution of the predicted values is similar to those observed. To guard against overfitting, we provide the same plots for the test set of 1049 children in Figs. 10 and 11. The same interpretation holds as for the training data: there are some minor deviations from the observed distribution, but overall the test set prediction of these distributions is quite accurate.

**Fig. 7:**
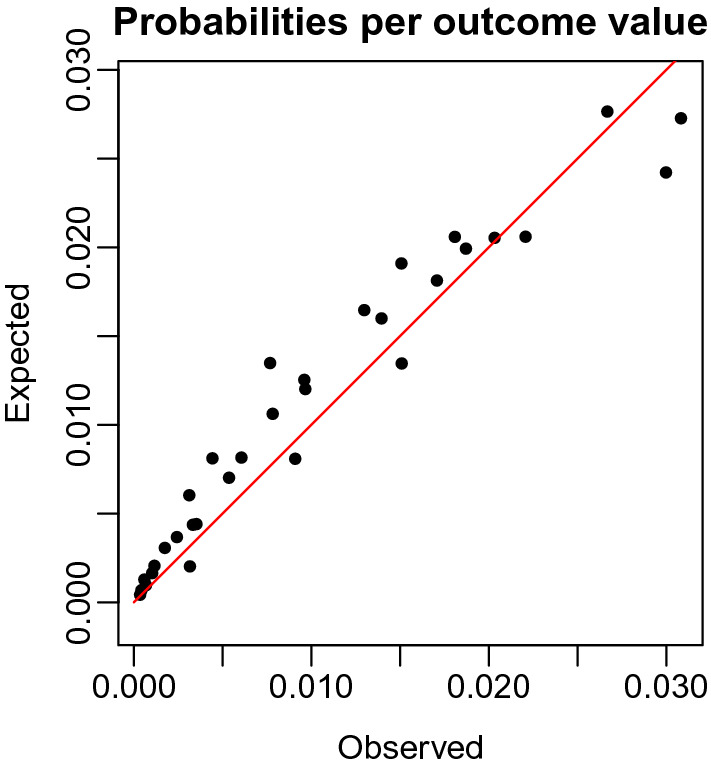
Scatterplot of the observed and expected probabilities per outcome value {0, 31}.

A widely used test to compare two distributions is the Chi-square goodness of fit test, which tests whether the observed sample is drawn from the predicted distribution. The in-sample Chi-square statistic is equal to $X^2 = 793.8$ ($p < 0.001$, $df = 31$). Note that only the uncensored observations are used to compute this statistic. It is well known that the Chi-square goodness of fit test is very sensitive to the number of observations (Cochran, 1952); therefore, we also look at the correlation between the observed and predicted probabilities. Figure 7 displays a strong correlation of 0.97 between the predicted and observed probabilities. The out-of-sample correlation is equal to 0.97. In addition, as a (dis)similarity measure we added the Hellinger distance between the predicted and observed distribution. The Hellinger distance is related to the Euclidean distance, so the closer the value to zero the similar the two distributions are. Both the in-sample and out-of-sample Hellinger distance is rounded equal to 0.08.

One of the model assumptions is conditional independence implying that any carry over effects are subsumed in the linear combination. To empirically justify this assumption, we additionally estimated the model with dummy variables up to ten lags whether or not a loss card was encountered. The strongest effect occurred immediately after the loss card was encountered. We found a monotonically decreasing effect of the lag of the loss trial, which can be seen as a support for the conditional independence assumption.

**Fig. 8:**
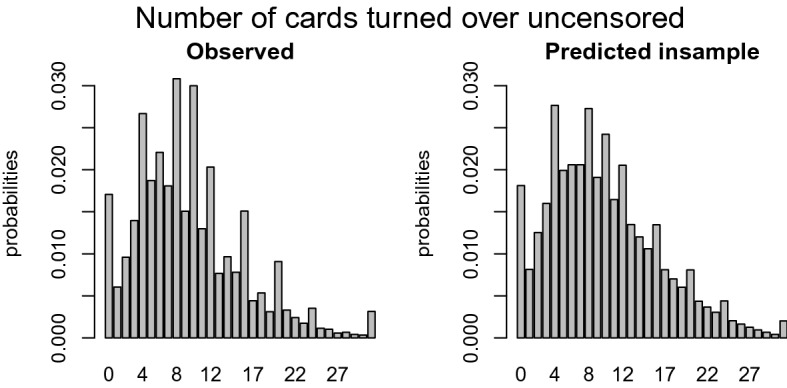
Distribution of the empirical (left panel) and predicted by the CMM (right panel) number of cards turned over for the uncensored observations in the training data.

**Fig. 9:**
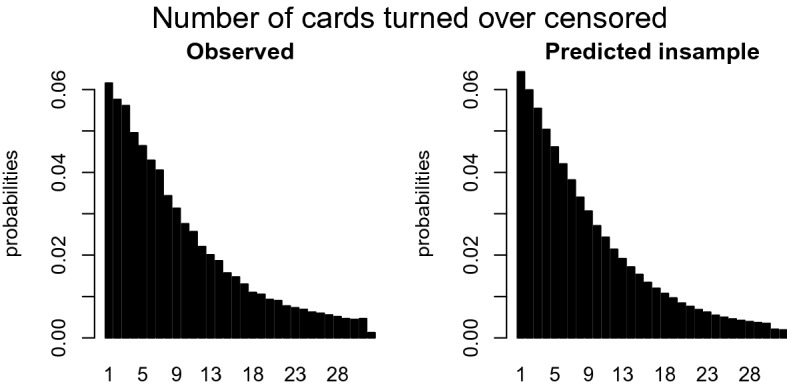
Distribution of the empirical (left panel) and predicted by the CMM (right panel) number of cards turned over corrected for the probability of being censored per card in the training data.

**Fig. 10:**
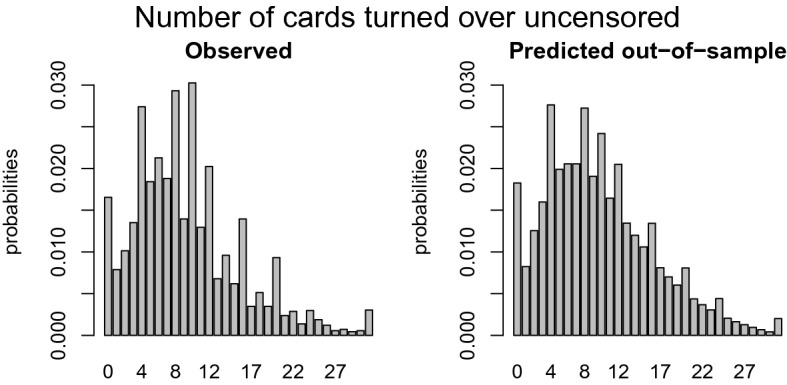
Distribution of the empirical (left panel) and predicted number of cards turned over by the CMM (right panel) for the uncensored observations in the test data.

**Fig. 11:**
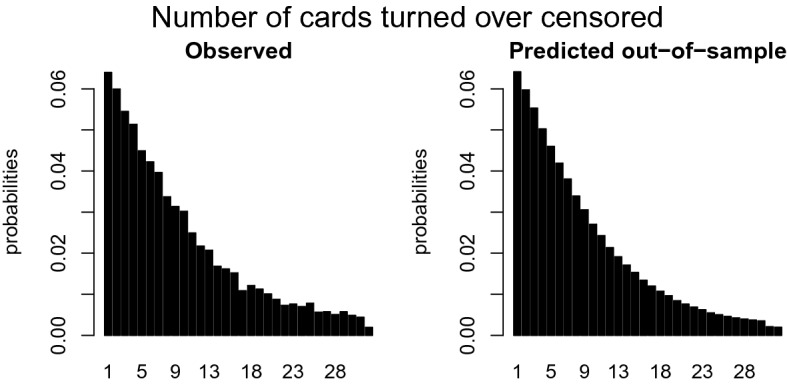
Distribution of the empirical (left panel) and predicted by the CMM (right panel) number of cards turned over corrected for the probability of being censored per card in the test data.

### Segment Specific Effects for Covariates

In addition to segment specific intercepts, the CMM also allows for segment specific effects for covariates. Here we discuss the results of a CMM with four segments and segment specific effects for the game settings gain amount, loss amount, and number of loss cards. The linear combination in this model becomes

$$\begin{aligned} \eta _{its} = \alpha _s + \tilde{\varvec{x}}_{it}' \tilde{\varvec{\beta }}_s + \varvec{x}{^{*}_{it}}' \varvec{\beta }^*, \end{aligned}$$

where $\tilde{\varvec{x}}_{it}'$ contains the game settings gain amount, loss amount, and number of loss cards, $\tilde{\varvec{\beta }}_s$ are the segment specific effects for these game settings, and $\varvec{x}{^{*}_{it}}'$ contains the covariates excluding the game setting variables. Note that we did not include interaction terms between the game settings and sex in this analysis, because these effects are captured by the segment specific effects.

The segment probabilities $\pi _s$, segment specific intercepts $\alpha _s$, and segment specific effects $\tilde{\varvec{\beta }}_s$ are presented in Table 5. The segment sizes and segment specific intercepts are comparable to the ones belonging to the CMM with four segments and only segments specific intercepts, see Table 2. It is clear that children in the fourth segment use to the information provided the most. In addition, the increase in information use is evident for all the game settings. So, these segments do not make a clear distinction between children who are, for example, more sensitive to reward than to punishment. Furthermore, children in the first segment only take into account the number of loss cards.

This model can be compared to the models including only segment specific intercepts by means of the BIC. The BIC for this model including segment specific effects for the game settings in addition to segment specific intercepts is equal to 133771. The BICs of the other models are presented in Fig. 5. In particular, the BIC value of 133909 for the four segments intercepts only model is slightly worse.

The other results from this analysis (i.e., regression coefficients, segment specific results, and model fit) are very similar to the ones presented above and, therefore, are included as Online Resource 2.

The four-segment model with segment specific effects for the game settings discussed in this section is just one of many that can be considered. In practice, various models can be compared that differ in the number of segments and the covariates that are segment specific. These models can be compared based on, for example, their BIC value.

**Table 5:** Results of the four segment CMM with both segment specific intercepts $\alpha _s$ and segment specific effects of the game setting parameters $\tilde{\varvec{\beta }}_s$.

	Segment *s*							
	1		2		3		4	
$\pi _s$	0.089	(0.027)	0.268	(0.072)	0.362	(0.068)	0.280	(0.041)
$\alpha _s$	5.239	(0.705)	10.409	(0.435)	17.914	(0.435)	35.127	(0.435)
Gain amount (10)	$-$0.043	(0.060)	0.327	(0.063)	1.347	(0.119)	1.351	(0.435)
Gain amount (30)	0.043	(0.060)	$-$0.327	(0.063)	$-$1.347	(0.119)	$-$1.351	(0.435)
Loss amount (250)	0.062	(0.059)	0.330	(0.063)	0.224	(0.110)	1.030	(0.435)
Loss amount (750)	$-$0.062	(0.059)	$-$0.330	(0.063)	$-$0.224	(0.110)	$-$1.030	(0.435)
Loss cards (1)	0.437	(0.062)	1.028	(0.065)	1.480	(0.117)	3.384	(0.464)
Loss cards (3)	$-$0.437	(0.062)	$-$1.028	(0.065)	$-$1.480	(0.117)	$-$3.384	(0.464)

The standard errors are given in parentheses.

### Parameter Recovery

We did a small simulation study to investigate the parameter recovery. This recovery is limited to the game setting parameters (i.e., the parameters belonging to the gain amount, loss amount and number of loss cards), as other predictors can be seen as covariates correcting the individual’s response for background variables. Data for $N = 500$ children who each played $T = 8$ trials are generated for two sets of game setting parameters $\beta _1$, $\beta _2$, and $\beta _3$. The data are sampled from $S = 2$ mixtures with segment specific intercepts $\alpha _1$ and $\alpha _2$. The probabilities $\phi _1$, $\phi _2$, $\phi _3$, and $\phi _4$ for the attractiveness for certain outcomes are set, such that they resemble the attractiveness in Fig. 2. Censoring is based on the probability of encountering a loss card as determined by the game setting of either one or three loss cards. In the simulation study, we use a slightly different parametrization from Appendix A to ensure uniqueness of the parameters. Particularly, we use $\alpha _1 = {\tilde{\alpha }}^2_1$ and $\alpha _2 = {\tilde{\alpha }}^2_1 + {\tilde{\alpha }}^2_2$ and optimize over ${\tilde{\alpha }}_1$ and ${\tilde{\alpha }}_2$. Without loss of generality, we enforce ${\tilde{\alpha }}_1 \ge 0$ and ${\tilde{\alpha }}_2 \ge 0$. These settings lead to a total of ten unique parameters, that is, $\beta _1$, $\beta _2$, $\beta _3$, ${\tilde{\alpha }}_1$, ${\tilde{\alpha }}_2$, $\delta$, $\tau _1$, $\tau _2$, $\tau _3$, and $\sigma$.

The definitions of the probabilities in the maximum likelihood function in the CMM served as the basis of the data generating process. For each of the parameter sets 500 replication data sets were drawn. Table 6 presents the two sets of true parameter values, the mean, median, and standard deviation of the estimated parameters, the average of the estimated standard errors (MeanSE), the RMSE, MAD, and coverage percentage of all model parameters. The RMSE, MAD, and coverage percentage are calculated with respect to the true parameter values and the coverage percentage is based on the 95% confidence intervals. For computational speed, we used the true parameter values as start values in the optimization process. The present recovery of the model parameters is good. Consistent with expectations when using 95% confidence intervals, almost all parameters in both sets have a coverage percentage around 95%. Furthermore, this parameter recovery allows us to investigate the performance of the BIC statistic in detecting the true number of segments. We compared the BIC statistic of the one segment models with the ones from the two segment models. In all replications in both sets, the two segment model is favored over the one segment model, thereby confirming the usefulness of the BIC statistic in identifying the number of segments.

**Table 6:** Recovery results of the CMM for two sets of true parameter values with $N = 500$ children each playing $T = 8$ trials.

Parameter	True value	Mean	Median	SD	MeanSE	RMSE	MAD	Coverage
*Set 1*								
$\beta _1$	2.00	1.98	1.96	0.34	0.35	0.34	0.27	0.95
$\beta _2$	$-$3.00	$-$3.02	$-$3.01	0.40	0.37	0.40	0.32	0.94
$\beta _3$	$-$6.00	$-$6.02	$-$5.98	0.44	0.42	0.44	0.35	0.95
${\tilde{\alpha }}_1$	3.39	3.39	3.39	0.09	0.08	0.09	0.07	0.94
${\tilde{\alpha }}_2$	2.92	2.92	2.91	0.10	0.10	0.10	0.08	0.96
$\delta$	3.00	3.03	3.02	0.21	0.22	0.22	0.17	0.96
$\tau _1$	$-$3.50	$-$3.51	$-$3.50	0.14	0.14	0.14	0.11	0.94
$\tau _2$	$-$1.90	$-$1.90	$-$1.90	0.15	0.15	0.15	0.12	0.95
$\tau _3$	$-$2.10	$-$2.12	$-$2.10	0.21	0.21	0.21	0.17	0.95
$\sigma$	$-$0.40	$-$0.40	$-$0.41	0.19	0.18	0.19	0.15	0.94
*Set 2*								
$\beta _1$	6.00	6.00	6.00	0.62	0.61	0.62	0.50	0.95
$\beta _2$	$-$4.00	$-$4.00	$-$3.99	0.56	0.54	0.55	0.45	0.95
$\beta _3$	$-$7.50	$-$7.55	$-$7.52	0.59	0.60	0.59	0.48	0.95
${\tilde{\alpha }}_1$	3.87	3.87	3.88	0.12	0.12	0.12	0.09	0.94
${\tilde{\alpha }}_2$	2.83	2.86	2.85	0.16	0.16	0.16	0.12	0.96
$\delta$	3.00	3.03	3.02	0.23	0.22	0.23	0.18	0.96
$\tau _1$	$-$3.50	$-$3.50	$-$3.50	0.13	0.13	0.12	0.10	0.96
$\tau _2$	$-$1.90	$-$1.90	$-$1.89	0.15	0.14	0.15	0.12	0.93
$\tau _3$	$-$2.10	$-$2.14	$-$2.10	0.30	0.29	0.30	0.23	0.96
$\sigma$	$-$0.40	$-$0.39	$-$0.41	0.39	0.35	0.39	0.30	0.91

Reported are the true parameter value, mean, median, and standard deviation (SD) of the estimated parameters, the average of the estimated standard errors (MeanSE), root mean square error (RMSE), mean absolute deviation (MAD), and coverage percentage.

## Discussion

The censored mixture model (CMM) is developed to solve three potential problems emerging with modeling risk taking and is applied to the Generation R data set, an exceptionally large data set with 3404 children that each completed 16 rounds of the Columbia Card Task (CCT). *First,* to accommodate the potential censoring that often occurs in sequential risk tasks, a cumulative distribution function is added to the likelihood function to compute the probability of turning over more cards than observed. In the Generation R data set, the prevalence of censoring is $68\%$. Ignoring the censoring would seriously underestimate the intended level of risk taking as more than two third of the data would not be used. *Second,* the inflated values in the outcome distribution are handled by assigning additional probability mass to these outcomes in the likelihood function. Figures 8, 9, 10, and 11 clearly show peaks at certain outcome values in both the observed and predicted graphs. Without the additional probability mass for the inflated values, the distributions in the predicted graphs would have been smoother and, hence, less similar to the observed graphs. *Finally*, four segments with a segment specific intercept are added to the model to account for unobserved heterogeneity across individuals. The distribution of posterior probabilities in Fig. 6 clearly points out that the four segments are distinctive as large probabilities (say above $80\%$) are most prevalent. In case the individuals in the sample all have the same tendency for risk taking, this graph would be centered around 0.25, indicating that the individuals are assigned to all segments with equal probability.

Similar challenges as with the CCT occur when analyzing risk taking through other sequential risk tasks, like the BART and Angling Risk Task. Hence, our CMM can also be used to analyze these risk tasks. Only minor adjustments to the model are necessary. For example, additional probability mass is assigned to inflated outcome values: for the CCT these values are $\{0, 4, 8, 10, 12, 16, 20, 24, 31\}$, which corresponds to creating geometric patterns, such as complete rows or columns. However, for other sequential risk tasks these values are likely to be different, depending upon the shape of the distribution of the outcome values and, hence, need to be adapted in the model.

We want to briefly discuss several elements of our CMM applied to our specific data set. The *selection of the number of segments* in the CMM was based on a three-way procedure: (a) the Bayesian information criterion (BIC) values of the different models are compared, (b) the segment specific intercepts had to be distinctive among the segments, and, (c) the smallest segment had to contain at least five percent of the sample. Although we are confident that a model with four segments is optimal in our case, a different strategy could have led to a different number of segments and, hence, slightly different results.

The CMM with four segments showed some interesting results in terms of *segment characteristics*. Both the most risk averse and risk seeking segments, respectively, Segments 1 and 4, have the highest level of behavioral problems measured by the child behavioral checklist (CBCL).

Furthermore, from Table 4, where the *regression coefficients* are shown it is clear that the number of loss cards has the highest effect on the number of cards turned over, compared to the gain amount and loss amount. This result is in accordance with many other studies using the CCT (Kluwe-Schiavon et al., 2015, Holper & Murphy, 2014, Penolazzi et al., 2012). Looking at the risk neutral strategy based on the expected values (Table 7), it is clear that turning over zero cards is often most profitable. It would be interesting to see whether different game settings lead to the same results. Note that we used the same game settings as (Figner & Weber, 2011). Additionally, children with a high IQ turn over fewer cards than the average and children from a family with a low household income turn over more cards than the average. Lastly, children with a Dutch or Asian background are more risk averse, compared to children with another ethnic background.

**Table 7:** Optimal number of cards to turn over when maximizing the expected value.

1 Loss card				3 loss cards			
		Loss amount				Loss amount	
		250	750			250	750
Gain amount	10	7	0	Gain amount	10	0	0
	30	23	6		30	4	0

We found a good *model fit* represented by a correlation of 0.97 between the observed and predicted probabilities of the number of cards at an aggregate level and a root mean square error (RMSE) of 8.5 and mean absolute deviation (MAD) of 5.5 derived from point estimates for the number of cards turned over. The out-of-sample correlation is equal to 0.97, the RMSE is equal to 8.4, and the MAD is equal to 5.3, showing little difference between in-sample and out-of-sample accuracy. Note that the current predictions are for unseen individuals. However, using a part of the trials for computing a posteriori segment probabilities would allow to make predictions for future trials taking better stock of the unobserved heterogeneity in the data.

Next to segment specific intercepts, the CMM also allows for *segment specific effects* for the game settings. We found that individuals in the most risk seeking segment, and thus with the highest segments specific intercept, also have the highest effects of the segment specific game setting weights. Furthermore, in all segments, the number of loss cards is taken into account. The difference between the segments lies in the use of the gain amount and loss amount.

A *parameter recovery* study showed a good recovery of the model parameters. Almost all parameters have a coverage percentage around 95%. Furthermore, this simulation study shows that the BIC is a useful statistic to identify the number of segments.

Recently, new light was shed on the debate concerning *parameter reliability*, that is, whether behavioral tasks provide reliable estimates and whether they have good construct validity. Pedroni et al. (2017) argue that people’s risk preferences are inconsistent across behavioral tasks. Holzmeister and Stefan (2021), meanwhile, provide evidence that participants are well aware of the variation in risk level associated with their choices and question the statement of inconsistent risk preferences. Note that the proposed CMM is not aimed at obtaining risk scores for individuals. Instead, our model provides useful conclusions on risk taking at the level of the entire sample.

It would also be interesting for further research to collect more information on the underlying decision process. For example, the time between actions (e.g., turning over cards) can be used to investigate a potential fatigue effect. In addition, more time between actions at the end of a trial could indicate that a participant doubts between continuing and stopping. Moreover, the pattern and sequence of the cards turned over could further justify our assumption that participants of the CCT create geometric patterns for turning over cards. Furthermore, the segment specific effects can be extended to other variables. For instance, the previous loss variables can be made segment specific to allow more freedom in capturing the carry-over effects.

A limitation of the current approach is that the negative binomial distribution has an infinite upper bound. This property implies that there is probability mass after 32, meaning that the model allows for turning over cards in a non-feasible region. However, all the point estimates (for computations, see Appendix C) are within the range $\{0, 32\}$. Therefore we argue that this is not a major issue. As an alternative to the negative binomial distribution, future studies can use a truncated version, that is, consider the negative binomial distribution conditional on the number of cards turned over being smaller or equal to 32 for the CCT. Taken together, we believe that the censored mixture model proposed in this paper is an important tool in the analysis of risk taking.

### Supplementary Information

Below is the link to the electronic supplementary material.

Supplementary file 1 (pdf 126 KB)

Supplementary file 2 (pdf 140 KB)

## Appendix A: Maximization of the Likelihood Function

To be able to maximize the likelihood over $\varvec{\theta }$, it is useful to have no constraints and to ensure that $\varvec{\theta }$ is unique. To do so, several reparametrizations are needed and we will represent that by the vector function $\varvec{h}()$. *First*, to ensure the restrictions $0 \le \phi _m \le 1$ and $\sum _{m=1}^4 \phi _m = 1$, we define

$$\begin{aligned} \varvec{\phi } = \varvec{h}_{\varvec{\phi }}\varvec{(\tau )} = \left\{ \begin{array}{ll} \exp (\tau _m)/(1 + \sum _{j = 1}^{3} \exp (\tau _j) ) &{} \text{ for } \ m \in \{1, 2, 3\} ,\\ 1 - \sum _{m=1}^{3} \exp (\tau _m)/(1 + \sum _{j = 1}^{3} \exp (\tau _j)) &{} \text{ for } \ m = 4. \end{array} \right. \end{aligned}$$

This reparametrization allows the constrained optimization over $\varvec{\phi }$ to be replaced by the unconstrained optimization over $\varvec{\tau }$. *Secondly*, a similar reparametrization is used for avoiding the sum one and nonnegativity constraints on the *S* segment probabilities in $\varvec{\pi }$ by $S - 1$ values through $\varvec{\pi } = \varvec{h_\pi (\sigma )}$.

*Thirdly*, there are sum zero constraints $\varvec{C}_j\varvec{\beta }_j = \varvec{0}$ on the weights corresponding to the dummy coding of the categories belonging to each categorical variable *j*. Instead of using these constraints, one of the categories per categorical variable is assigned as a reference category and consequently that particular weight in $\varvec{\beta }_j$ is set to zero, effectively excluding these weights from the optimization. This implies that the effect of the reference categories is included in the intercepts $\varvec{\alpha }_u$. For notational convenience, it is useful to gather the intercepts in $\varvec{\alpha }$ and weights $\varvec{\beta }$ into a single vector $\varvec{\gamma }$, that is, $\varvec{\gamma }_u = [\varvec{\alpha }'_u, \varvec{\beta }'_u]'$ where $\varvec{\beta }_u$ is the vector of weights with the values corresponding to reference categories fixed to zero. The transformation needed from the unique vector of parameters $\varvec{\gamma }_u = [\varvec{\alpha }'_u, \varvec{\beta }'_u]'$ to the non-unique vector $\varvec{\gamma } = [\varvec{\alpha }', \varvec{\beta }']'$ is illustrated by the following example with $S = 3$, two categorical variables, and two numerical variables. Then,

$$\begin{aligned} \varvec{\gamma } = \left[ \begin{array}{c} \varvec{\alpha } \\ \varvec{\beta }_1 \\ \varvec{\beta }_2 \\ \beta _{3}\\ \beta _{4} \end{array} \right] = \varvec{h}_{\varvec{\gamma }}(\varvec{\gamma }_u) = \left[ \begin{array}{ccccc} \varvec{I} &{}\quad K_1^{-1}\varvec{11}'&{}\quad K_2^{-1}\varvec{11}' &{}\quad \varvec{0} &{}\quad \varvec{0} \\ \varvec{0} &{}\quad \varvec{I} - K_1^{-1}\varvec{11}' &{}\quad \varvec{0} &{}\quad \varvec{0} &{}\quad \varvec{0}\\ \varvec{0} &{}\quad \varvec{0} &{}\quad \varvec{I} - K_2^{-1}\varvec{11}' &{}\quad \varvec{0} &{}\quad \varvec{0}\\ \varvec{0} &{}\quad \varvec{0} &{}\quad \varvec{0} &{} 1 &{}\quad 0\\ \varvec{0} &{}\quad \varvec{0} &{}\quad \varvec{0} &{}\quad 0&{}\quad 1\\ \end{array} \right] \left[ \begin{array}{c} \varvec{\alpha }_u \\ \varvec{\beta }_{u1} \\ \varvec{\beta }_{u2} \\ \beta _{u3}\\ \beta _{u4} \end{array} \right] = \varvec{A\gamma }_u, \end{aligned}$$

where $K_j$ is the number of categories for categorical variable *j* and the matrices $\varvec{I}, \varvec{0}$, and $\varvec{11}'$ are understood to be adapted to the corresponding sizes depending on the relevant lengths of the vectors $\varvec{\alpha }_u$ and $\varvec{\beta }_{uj}$.

Let $\varvec{\theta }_u '= [\varvec{\gamma }_u', \delta , \varvec{\tau }', \varvec{\sigma }']$ be the unconstrained vector of all uniquely defined parameters. Then, the reparametrization function $\varvec{h}(\varvec{\theta }_u)$ can be written as

$$\begin{aligned} \varvec{\theta } = \varvec{h}(\varvec{\theta }_u) = [\varvec{h}_{\varvec{\gamma }}'(\varvec{\gamma }_u), \delta , \varvec{h}_{\varvec{\phi }}'(\varvec{\tau }), \varvec{h}_{\varvec{\phi }}'(\varvec{\sigma })]'. \end{aligned}$$

Thus, the constrained maximization of $\varvec{\theta }$ in (1) is equivalent to the unconstrained maximization

$$\begin{aligned} L(\varvec{\theta }) = L(\varvec{h}(\varvec{\theta }_u) = L_u(\varvec{\theta }_u). \end{aligned}$$

To maximize $L_u(\varvec{\theta }_u)$ over $\varvec{\theta }_u$, we use the BFGS quasi-Newton algorithm as implemented in the optimx package in R. After convergence, one step of the Newton-Raphson method with a numerically approximated Hessian is performed to ensure the gradient to be close to zero.

It is well known that for maximum likelihood estimation, at a maximum $\varvec{\theta }_u^*$, the parameters are normally distributed $\varvec{\theta }_u \sim N(\varvec{\theta }_u^*, \varvec{\Sigma }_u)$ where $\varvec{\Sigma }_u = -(\nabla ^2 L_u(\varvec{\theta }_u^*)^{-1})$ is the inverse of the negative Hessian of $L_u()$ evaluated at $\varvec{\theta }^*_u$. As we would like to have the covariance matrix $\varvec{\Sigma }_{\varvec{\theta }}$ of $\varvec{\theta }^* = \varvec{h}(\varvec{\theta }_u^*)$, the Delta method is applied so that

2 $$\begin{aligned} \varvec{\theta }^* \sim N(\varvec{h}(\varvec{\theta }_u), \varvec{\Sigma }_{\varvec{\theta }} ) \end{aligned}$$

where $\varvec{\Sigma }_{\varvec{\theta }} = \nabla \varvec{h}'(\varvec{\theta }_u)\varvec{\Sigma }_u \nabla \varvec{h}(\varvec{\theta }_u)$ and $\nabla \varvec{h}(\varvec{\theta }_u)$ is the Jacobian matrix of $\varvec{h}()$, that is,

$$\begin{aligned} \nabla \varvec{h}(\varvec{\theta }_u) = \left[ \begin{array}{cccc} \nabla \varvec{h}_{\varvec{\gamma }}(\varvec{\gamma }_f^*) &{}\quad 0 &{}\quad \varvec{0} &{}\quad \varvec{0} \\ \varvec{0} &{}\quad 1 &{}\quad \varvec{0} &{}\quad \varvec{0} \\ \varvec{0} &{}\quad 0 &{}\quad \nabla \varvec{h}_{\varvec{\phi }}(\varvec{\tau }_u^*) &{}\quad \varvec{0} \\ \varvec{0} &{}\quad 0 &{}\quad \varvec{0} &{}\quad \nabla \varvec{h}_{\varvec{\pi }}(\varvec{\sigma }_u^*) \\ \end{array} \right] , \end{aligned}$$

where $\nabla \varvec{h}_{\varvec{\gamma }}(\varvec{\gamma }_f^*) = \varvec{A}$, $\nabla \varvec{h}_{\varvec{\phi }}(\varvec{\tau }_u^*)$, and $\nabla \varvec{h}_{\varvec{\pi }}(\varvec{\sigma }_u^*)$ are the Jacobians for $\varvec{h}_{\varvec{\gamma }}()$, $\varvec{h}_{\varvec{\phi }}()$, and $\varvec{h}_{\varvec{\pi }}()$, respectively. The standard errors of the model parameters in $\varvec{\theta }$ are the square roots of the diagonal elements of the covariance matrix in (2).

## Appendix B: Significance Testing for Weighted Means

Obtaining a test for the differences between weighted means as presented in Table 3, some non-standard steps are needed. Let $\varvec{P}$ be the $n \times S$ matrix of the a posteriori probabilities from the CMM. Then, regress the a posteriori probabilities in $\varvec{P}$ on a CBCL symptom subscale without intercept through OLS. The obtained regression coefficients $\varvec{\psi }$ can be transformed to the weighted averages as follows:

$$\begin{aligned} \varvec{\psi }^* = \varvec{B}\varvec{\psi }, \text { with } \varvec{B} = \text {Diag}(\varvec{P}'\varvec{1})^{-1} \varvec{P}'\varvec{P}, \end{aligned}$$

where the operator Diag(.) transforms a vector into a diagonal matrix. We can do a Wald test with null hypothesis that the weighted means are the same ($\varvec{\psi }^* = c\varvec{1}$). The standard errors needed for the Wald test can be derived from the diagonal elements of the covariance matrix $\varvec{\Sigma }_{\varvec{\psi }^*} = \varvec{B}'\varvec{\Sigma _{\varvec{\psi }}} \varvec{B}$, where $\varvec{\Sigma _{\varvec{\psi }}}$ is the original covariance matrix obtained from the linear regression.

## Appendix C: CMM Expected Values

As the CMM provides a probability distribution for each number of cards turned over on each trial, obtaining predictions from a censored mixture model (CMM) is not straightforward. Therefore, we choose the expectation as a point estimate for the predicted value. These expectations can be obtained from the following steps. First, the estimated regression coefficients ${\hat{\alpha }}_s$ and ${\hat{\beta }}$ are used to compute the linear combination

$$\begin{aligned} {\hat{\mu }}_{its} = \log (\exp ({\hat{\alpha }}_s + \varvec{x}'_{it}\varvec{{\hat{\beta }}}) +1). \end{aligned}$$

Second, for each individual, in each trial, and for each segment we can compute the probabilities of all possible outcomes $y \in \{0,\ldots , 32\}$,

3 $$\begin{aligned} \Pr (Z_{it} = \ell \mid {\hat{\mu }}_{its}, {\hat{\delta }}) = \left\{ \begin{array}{ll} {\hat{\phi }}_4 f(0 \mid {\hat{\mu }}_{its}, {\hat{\delta }}) + \hat{\phi _1} &{} \text {if } \ell = 0,\\ {\hat{\phi }}_4 f(\ell \mid {\hat{\mu }}_{its}, {\hat{\delta }}) + \frac{1}{|A|} \hat{\phi _2} &{} \text {if } \ell \in A,\\ {\hat{\phi }}_4 f(31 \mid {\hat{\mu }}_{its}, {\hat{\delta }}) + \hat{\phi _3} &{} \text {if } \ell = 31,\\ {\hat{\phi }}_4 f(32 \mid {\hat{\mu }}_{its}, {\hat{\delta }}) &{} \text {if } \ell = 32,\\ {\hat{\phi }}_4 f(\ell \mid {\hat{\mu }}_{its}, {\hat{\delta }}) &{} \text {for all other values } \ell , \end{array} \right. \end{aligned}$$

where $f(\ell \mid {\hat{\mu }}_{its}, {\hat{\delta }})$ is the probability mass function of the negative binomial distribution, $A = \{4, 8, 10, 12, 16, 20, 24\}$, |*A*| is the cardinality of set *A*, and ${\hat{\phi }}_j$ is the estimate of $\phi _j$. The distributions in Figures 8, 9, 10, and 11 are obtained by weighting for the segments, using the estimated prior segment probability ${\hat{\pi }}_s$, and then summing over the individuals, trials, and segments

$$\begin{aligned} h_{it}(y) = \sum _{i = 1}^{N} \sum _{t=1}^{T} \sum _{s = 1}^{S} {\hat{\pi }}_s \Pr (Z_{it} = \ell \mid {\hat{\mu }}_{its}, {\hat{\delta }}) \ \ \ \ \forall \ \ell \in \{0,\ldots , 32\}. \end{aligned}$$

Note that the right graphs of these figures are multiplied by the probability of being (un)censored to get the same scale as the left graph.

The expected values per person and per trial can be obtained from the probabilities in (3), that is,

$$\begin{aligned} {\hat{y}}_{it} = \sum _{s = 1}^{S} {\hat{\pi }}_s \left( \sum _{\ell = 0}^{M} \ell \Pr (Z_{it} = \ell \mid {\hat{\mu }}_{its}, {\hat{\delta }}) + M{\hat{\phi }}_1(1-F(M+1 \mid {\hat{\mu }}_{its}, {\hat{\delta }}))\right) . \end{aligned}$$

The probability mass above *M*, denoted by $1-F(M+1 \mid {\hat{\mu }}_{its}, {\hat{\delta }})$, is added to the expected values as if it were the probability mass at *M*. Since the negative binomial distribution has an infinite upper bound, *M* should be large to obtain an accurate expected value. The probability mass above $M = 100$ is smaller than 0.0001 and so will not have a meaningful effect on the expected value. Therefore, we choose $M = 100$. Again, the expected values are multiplied by the probability of being uncensored to allow for a fair comparison with the observed outcome.

## Footnotes

1 At the beginning of the data collection we decided to shorten the test. Instead of three blocks with in total 24 trials, two blocks with in total 16 trials were played. There are 388 children, who played 24 trials. For the analysis only the first 16 trials of these children are used.
