# Supplementary material for: A Censored Mixture Model for Modeling Risk Taking
Source: Psychometrika. 2022 Feb 10;87(3):1103–29. doi: 10.1007/s11336-021-09839-1 (PMC9433365; doi:10.1007/s11336-021-09839-1)
Supplement: Supplementary file 1 — (pdf 126 KB) [file 11336_2021_9839_MOESM1_ESM.pdf]

A Censored Mixture Model for Modeling Risk Taking - Supplementary Material Censored Mixture Model Applied to BART

February 2, 2022

Censored Mixture Model applied to the Balloon Analogue Risk Task

The Censored Mixture Model (CMM) can be more generally applied than only to the Columbia Card Task (CCT). To show this, we have also analysed the Balloon Analogue Risk Task (BART) with the CMM. In this supplementary material we shall apply the CMM to a small data set and report the fit measures and overall distributions.

The data used in this supplementary study is provided by Dekkers et al. (2020). The data contains 180 boys, aged 12-19 years old, and 81 adolescents have ADHD. Next, we have information about the Social Economic Status (SES), IQ, and ethnicity (Dutch 76.7%, other western 55.6%, and non western 17.8%). Additionally, we use the variables previous loss and second previous loss as predictors. These variables indicate whether the balloon in the (second) previous trial exploded or not. This leads to a total of $12 + 2S - 1$ ($= S$ segment specific intercepts $\boldsymbol{\alpha}$ + 8 regression coefficients $\boldsymbol{\beta}$ + 1 scale parameter δ + 3 weight parameters $\boldsymbol{\tau}$ + $S - 1$ segment probabilities $\boldsymbol{\sigma}$) estimators that need to be estimated in a model with S segments. Every participant played the BART thirty times, meaning that we

have 5400 (= 180 adolescents $*$ 30 trials) observations to estimate these parameters. More information about the data collection can be found in Dekkers et al. (2020).

Similar as with the Columbia Card Task (CCT), the BART has certain outcomes that are more attractive than others. We distinguish four categories: (a) $k = 50$, (b) multiples of ten except for 50 (i.e., $k \in A$ with $A = \{10, 20, 30, 40, 60, 70, 80, 90, 100\}$), (c) multiples of five that are not multiples of ten (i.e., $k \in B$ with $B = \{5, 15, 25, 35, 45, 55, 65, 75, 85, 95\}$), and (d) otherwise. The same computation settings as in the Section 5 are used. Note that we did not split the data in a training and test set, and therefore have no out-of-sample performance measures, because the data set is too small.

The Bayesian information criterion (BIC) is lowest for the model with five segments, see Figure 1. Although the first segment in the model with five segments is small, see Table 1, we believe that the difference between the segment specific intercepts α_s, in particular the difference between the first segment and the other segments, are relevant. Therefore, we choose the model with five segments. Table 2 displays the game characteristics weighted by the a posteriori probabilities per segment. The stars denote that for all characteristics at least one of the segment averages is significantly different from the overall average. Furthermore, the regression coefficients are presented in Table 3. Similar as in the CCT, the results from the previous and second previous trial have a large impact on the behavior in the current trial. Last, we found a reasonable good correlation of 0.92, Figure 3, with an RMSE of 17.8 and MAD of 15.4. Figure 2 displays the distributions of the empirical and predicted number of pumps for the uncensored observations. The Hellinger distance between these two distributions is 0.27.

Table 1: Segment probabilities π_s and segment specific intercepts α_s, with the standard errors between brackets for CMMs with S = 2 to 6 segments.

S		Segment s 1	2	3	4	5	6
2							
	π_s	0.324	0.676				
		(0.042)	(0.042)				
	α_s	35.696	65.563				
		(1.614)	(2.065)				
3							
	π_s	0.112	0.323	0.565			
		(0.028)	(0.042)	(0.046)			
	α_s	24.125	45.88	70.347			
		(1.394)	(1.799)	(1.777)			
4							
	π_s	0.062	0.301	0.456	0.181		
		(0.019)	(0.040)	(0.051)	(0.044)		
	α_s	12.445	33.921	55.52	78.583		
		(1.895)	(1.820)	(2.101)	(3.772)		
5							
	π_s	0.028	0.094	0.251	0.533	0.095	
		(0.012)	(0.024)	(0.041)	(0.054)	(0.045)	
	α_s	2.298	24.893	41.335	59.375	87.706	
		(1.862)	(1.696)	(1.478)	(1.947)	(8.790)	
6							
	π_s	0.028	0.094	0.246	0.479	0.081	0.073
		(0.012)	(0.024)	(0.041)	(0.155)	(0.139)	(0.047)
	α_s	2.147	24.801	41.213	58.36	69.333	90.971
		(1.901)	(1.763)	(1.536)	(2.304)	(16.324)	(8.423)

Table 2: Weighted scores per segment of game characteristics.

	Segment s 1	2	3	4	5	Total
π_s	0.03	0.09	0.25	0.53	0.09	
Average score ***	21.3	22.0	26.8	28.3	26.7	27.0
# pumps ***	30.8	28.6	38.6	45.5	49.4	42.1
# censored trials ***	7.8	6.3	10.1	13.4	15.8	12.0

A Wald test is performed to check for a significant difference between the segments. Three stars denotes $p < 0.01$.

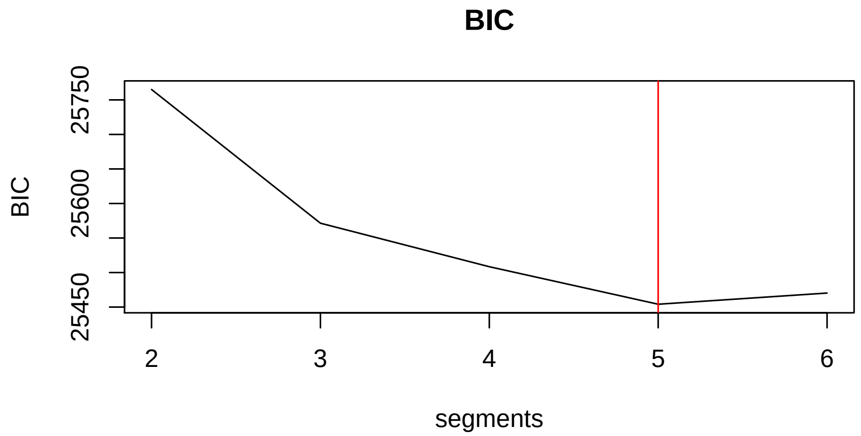

Figure 1: The Bayesian information criterion (BIC) of CMMs with $S = 2$ to 6 segments in the BART.

Table 3: Regression coefficients with their standard errors. Within a categorical variable the sum of coefficients sum to zero and the continues variables IQ, Social Economic Status, and age are standardized.

	β-coefficients	(st error)
IQ	0.547	(0.616)
Social Economic Status	0.743	(0.811)
Age	1.275	(0.492)
Previous loss yes	-2.899	(0.420)
Previous loss no	2.899	(0.420)
Second previous loss yes	-1.163	(0.444)
Second previous loss no	1.163	(0.444)
Ethnicity		
Western	3.146	(2.866)
Non Western	-1.714	(1.617)
Dutch	-1.432	(1.411)
ADHD group	0.046	(0.478)
Control group	-0.046	(0.478)

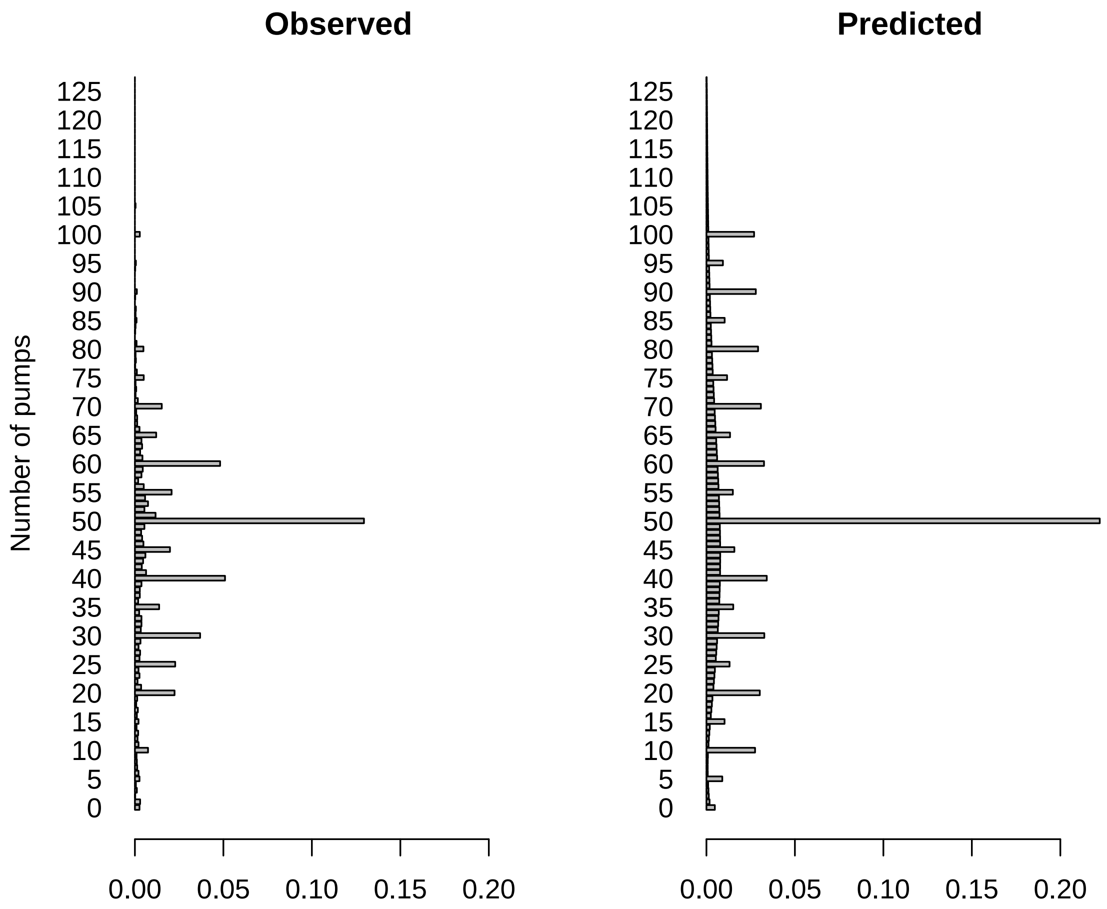

Figure 2: Distribution of the empirical (left panel) and predicted by the CMM (right panel) number of pumps for the uncensored observations in the BART.

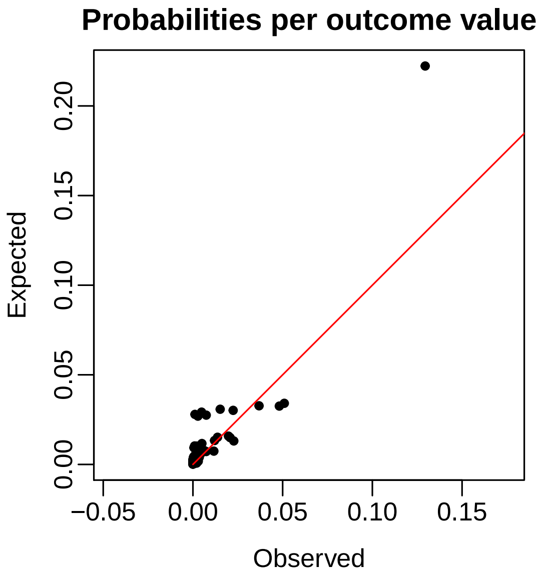

Figure 3: Scatterplot of the observed and expected probabilities per outcome value $\{0, 127\}$
