# Supplementary material for: A Censored Mixture Model for Modeling Risk Taking
Source: Psychometrika. 2022 Feb 10;87(3):1103–29. doi: 10.1007/s11336-021-09839-1 (PMC9433365; doi:10.1007/s11336-021-09839-1)
Supplement: Supplementary file 2 — (pdf 140 KB) [file 11336_2021_9839_MOESM2_ESM.pdf]

A Censored Mixture Model for Modeling Risk Taking - Supplementary Material Segment Specific Effects for Game Settings

February 2, 2022

Censored Mixture Model with Segment Specific Effects for the Game Setting Variables

In addition to segment specific intercepts, the CMM also allows for segment specific effects for covariates. Here we present results of a CMM with four segments and segment specific effects for the game settings gain amount, loss amount, and number of loss cards. The linear combination in this model becomes

$$\eta_{its} = \alpha_s + \tilde{\boldsymbol{x}}_{it}'\tilde{\boldsymbol{\beta}}_s + \boldsymbol{x}_{it}^{*\prime}\boldsymbol{\beta}^*,$$

where $\tilde{\boldsymbol{x}}_{it}'$ contains the game settings gain amount, loss amount, and number of loss cards, $\tilde{\boldsymbol{\beta}}_s$ are the segment specific effects for these game settings, and $\boldsymbol{x}_{it}^{*\prime}$ contains the covariates excluding the game setting variables. Note that we did not include interaction terms between the game settings and sex in this analysis, because these effects are captured by the segment specific effects.

The segment specific effects for the game settings $\tilde{\boldsymbol{\beta}}_s$ are, next to the segment specific intercept α_s and segment probabilities π_s, presented in Section 5.5. The other results,

presented in this supplementary material, are very similar to the results from the analysis with only segment specific intercepts, discussed in Section 5.

The regression coefficients from Table 1 have a linear interpretation, because our link function is close to the identity function, see Figure 4 in Section 4. Also, the numerical variables (age and IQ) are standardized to z-scores prior to the analysis and the categorical variables are constrained to have mean weights of the categories belonging to a single variable equal to zero, so that the intercept can be interpreted as the average score in the segment for a neutral child. Similar to the analysis with only segment specific intercepts, girls take more risk than boys and IQ is negatively associated with the number of cards turned over. Also, a household income between 2000 and 4000 euro's per month is related to lower risk taking levels. Children with a mother with a Dutch or Dutch Antilles ethnicity turn over fewer cards than the base average and children with a mother with an African, Moroccan, or Turkish ethnicity turn over more cards than the base average. Furthermore, children with a mother who had no or primary education take more risk than children with a mother with a higher education. Moreover, the results from the previous round have a strong effect on observed behavior in the current round. In case a loss card was encountered in the previous or second previous round on average 1.6 and 1 cards less are turned over, respectively.

Again, the risk averse children in Segment 1 and the risk seekers in Segment 4 on average have more behavioral problems than the children in Segment 2 and 3. Particularly, the risk averse children are more anxious and aggressive. The risk seekers, on the other hand, have more attention problems and show more delinquent behavior than the children in other segments.

Last, we found a reasonable good correlation of 0.97, see Figure 1, with an RMSE of 8.5 and MAD of 5.5. Figures 2 and 3 display the distributions of the empirical and predicted

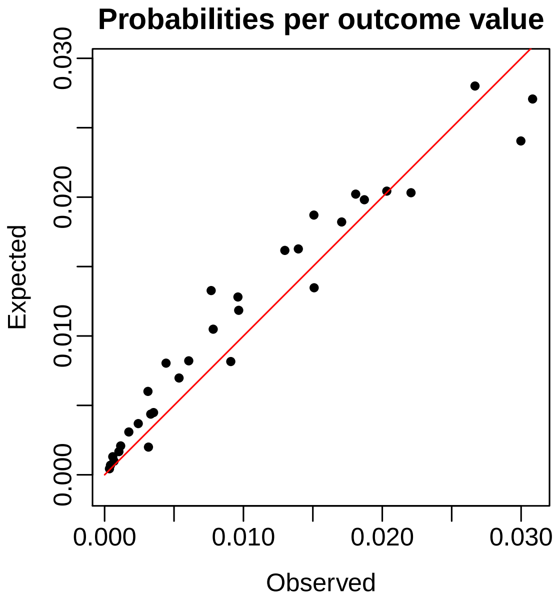

Figure 1: Scatterplot of the observed and expected probabilities per outcome value {0, 31} for the model with segment specific effects for the game setting variables.

number of cards turned over for the uncensored and censored observations, respectively. The Hellinger distance between the observed and predicted distribution of the uncensored cases is equal to 0.08. To guard against overfitting, we provide the same performance measures for the test set containing 1049 children. The out-of-sample RMSE is equal to 8.4, the MAD is equal to 5.4, and the correlation is equal to 0.97. The distributions of the empirical and predicted out-of-sample number of cards turned over for the uncensored and censored observations are presented in Figures 4 and 5, respectively. The out-of-sample Hellinger distance equals 0.08.

Table 1: Regression coefficients with their standard errors from the model with segment specific effects for the game setting variables. Within a categorical variable the sum of coefficients sum to zero and the continues variables age and IQ are standardized.

	β - coefficients	(st error)
Age	-0.105	(0.062)
Boy	-0.310	(0.072)
Girl	0.310	(0.072)
IQ	-0.350	(0.089)
Ethnicity mother		
Dutch	-0.686	(0.142)
Asian	-0.047	(0.231)
African	1.408	(0.349)
Moroccan	0.604	(0.301)
Dutch Antilles	-3.263	(0.349)
Surinamese	0.429	(0.277)
Turkish	1.155	(0.303)
Other Western	0.398	(0.247)
Education mother		
No or primary education	0.543	(0.193)
Secondary education	-0.206	(0.124)
Higher education	-0.337	(0.125)
Householdincome per month in euro's		
< 2000	0.133	(0.132)
$2000 - 4000$	-0.284	(0.100)
> 4000	0.151	(0.111)
Previous loss yes	-0.820	(0.039)
Previous loss no	0.820	(0.039)
Second previous loss yes	-0.489	(0.039)
Second previous loss no	0.489	(0.039)

Table 2: Weighted z-scores per segment of CBCL subscales scores and other CCT characteristics for the model with segment specific effects for the game settings.

	Segment s				
	1	2	3	4	Total
π_s	0.09	0.27	0.36	0.28	
CBCL subscales					
Internalizing *	0.08	-0.01	-0.05	0.05	0.00
Externalizing **	0.09	-0.04	-0.04	0.06	0.00
CBCL symptom scales					
Anxiety **	0.10	0.00	-0.05	0.04	0.00
Social withdrawl **	0.02	0.02	-0.06	0.05	0.00
Somatic complaints	0.06	-0.03	0.00	0.02	0.00
Social problems ***	0.11	-0.05	-0.06	0.09	0.00
Thought problems **	0.11	-0.02	-0.06	0.06	0.00
Attention problems ***	-0.01	-0.05	-0.06	0.13	0.00
Delinquent behavior **	0.00	-0.04	-0.03	0.08	0.00
Aggressive behavior **	0.12	-0.03	-0.04	0.05	0.00
Average score ***	-87.6	-120.8	-170.0	-228.1	-165.7
# cards turned over ***	4.9	7.5	9.9	11.7	9.3
# censored trials ***	5.7	8.4	11.3	14.3	10.8

A Wald test is performed to check for a significant difference between the segments. One star denotes $0.05 \leq p < 0.10$, two $0.01 \leq p < 0.05$, and three $p < 0.01$. The 223 children without a CBCL score measured at either six or nine years old were excluded.

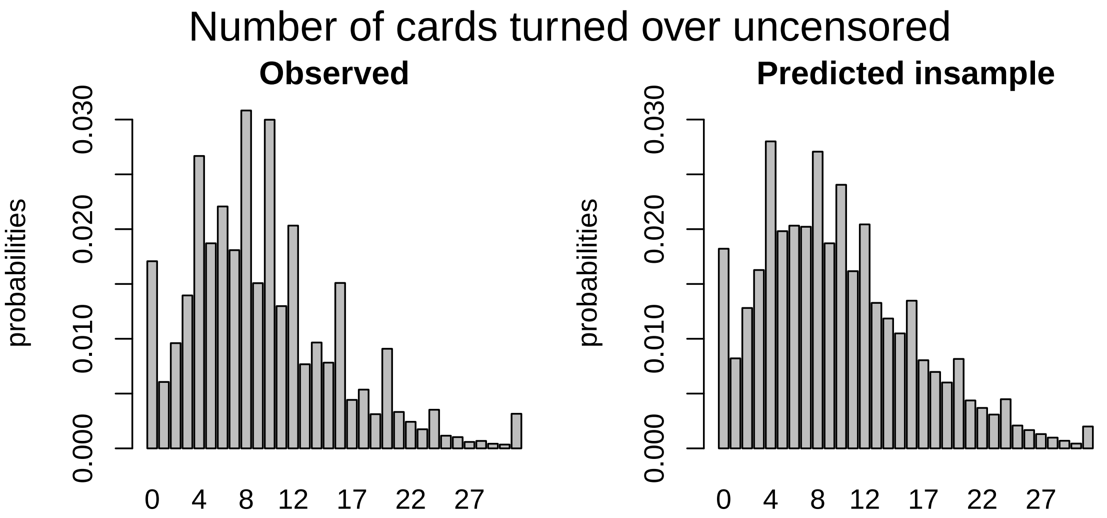

Figure 2: Distribution of the empirical (left panel) and predicted by the CMM with segment specific effects for the game settings (right panel) number of cards turned over for the uncensored observations in the training data.

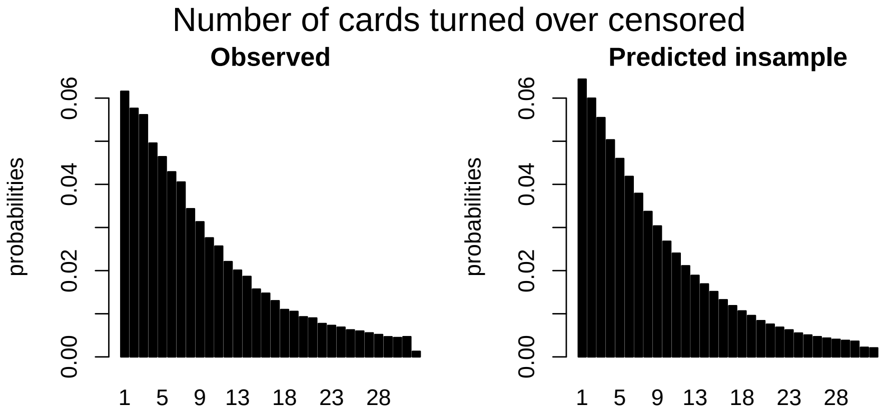

Figure 3: Distribution of the empirical (left panel) and predicted by the CMM with segment specific effects for the game settings (right panel) number of cards turned over corrected for the probability of being censored per card in the training data.

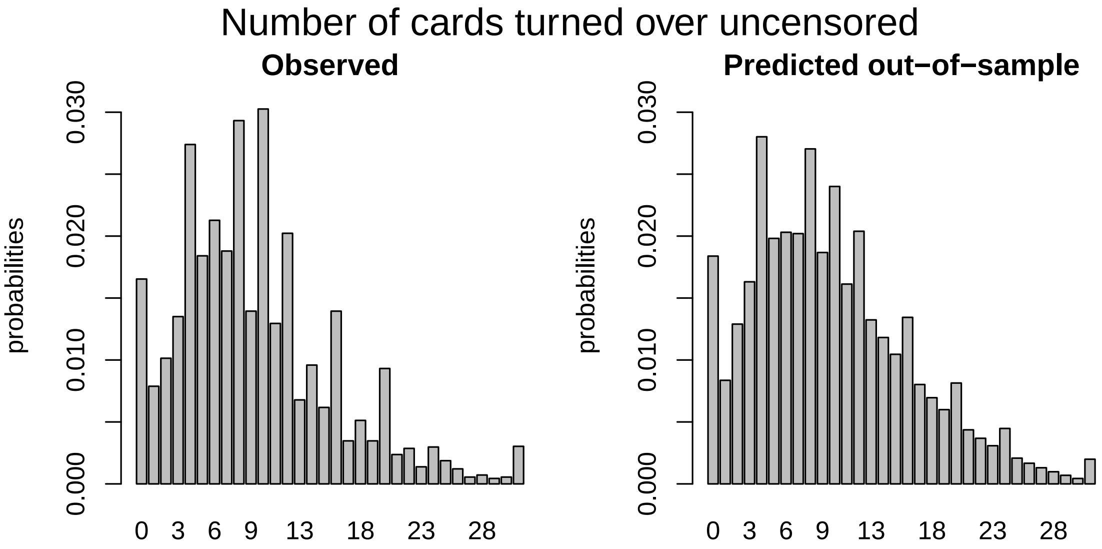

Figure 4: Distribution of the empirical (left panel) and predicted by the CMM with segment specific effects for the game settings (right panel) number of cards turned over for the uncensored observations in the test data.

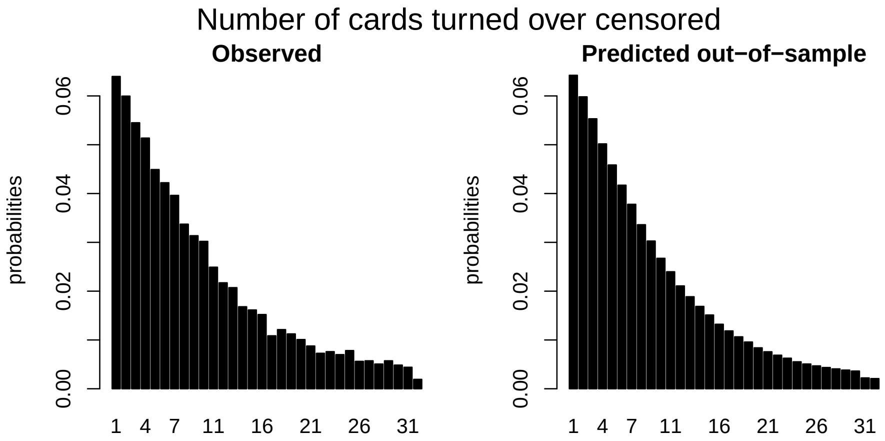

Figure 5: Distribution of the empirical (left panel) and predicted by the CMM with segment specific effects for the game settings (right panel) number of cards turned over corrected for the probability of being censored per card in the test data.
